# *Escherichia coli* Flagellar Genes as Target Sites for Integration and Expression of Genetic Circuits

**DOI:** 10.1371/journal.pone.0111451

**Published:** 2014-10-28

**Authors:** Mario Juhas, Lewis D. B. Evans, Joe Frost, Peter W. Davenport, Orr Yarkoni, Gillian M. Fraser, James W. Ajioka

**Affiliations:** Department of Pathology, University of Cambridge, Cambridge, United Kingdom; Imperial College London, United Kingdom

## Abstract

*E. coli* is a model platform for engineering microbes, so genetic circuit design and analysis will be greatly facilitated by simple and effective approaches to introduce genetic constructs into the *E. coli* chromosome at well-characterised loci. We combined the Red recombinase system of bacteriophage λ and Isothermal Gibson Assembly for rapid integration of novel DNA constructs into the *E. coli* chromosome. We identified the flagellar region as a promising region for integration and expression of genetic circuits. We characterised integration and expression at four candidate loci, *fliD, fliS, fliT,* and *fliY,* of the *E. coli* flagellar region 3a. The integration efficiency and expression from the four integrations varied considerably. Integration into *fliD* and *fliS* significantly decreased motility, while integration into *fliT* and *fliY* had only a minor effect on the motility. None of the integrations had negative effects on the growth of the bacteria. Overall, we found that *fliT* was the most suitable integration site.

## Introduction

**Figure 1:**
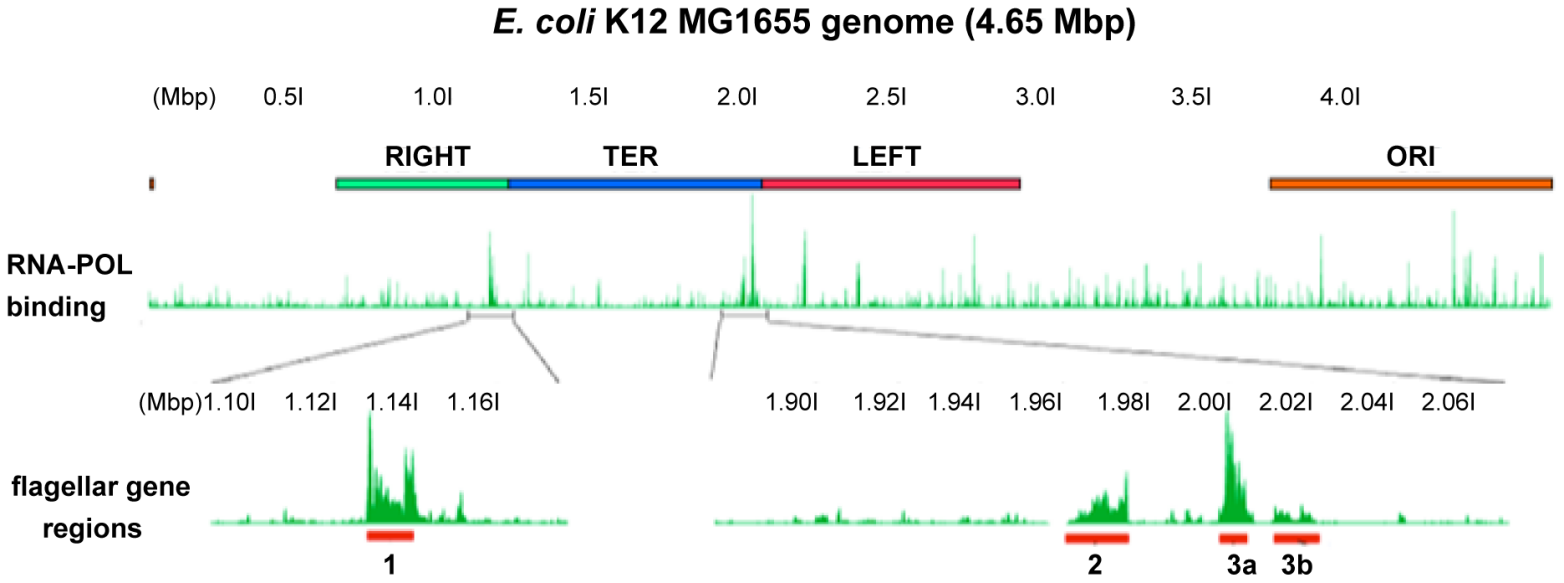
Location and expression of the *E. coli* flagellar regions. The *E. coli* K12 MG1655 genome showing positions of macrodomains (RIGHT, TER, LEFT, and ORI) and RNA polymerase (RNA-POL) binding (green peaks; ChIP-seq data from cells at mid-exponential growth phase [10]). *E. coli* flagellar genes are among the top 0.5% most highly expressed genes during exponential growth. The finding that the flagellar genes are among the top 0.5% of highly expressed genes in E. coli was derived from the RNA-Pol binding data obtained by Kahramanoglou *et al* [10]. Figure was generated by uploading the RNA-Pol binding data to the UCSC microbial genome browser for *E. coli* K12 MG1655 (http://microbes.ucsc.edu/cgi-bin/hgGateway?db=eschColi_K12).

The Gram-negative rod-shaped bacterium *Escherichia coli* K-12 is one of the most common microbes used for bioproduct manufacturing, metabolic engineering and as a chassis for Synthetic Biology devices [1]–[5]. Introduction of novel genes/genetic circuits into *E. coli* on plasmids has many disadvantages, such as increased metabolic burden, variable copy numbers and the necessity for constant antibiotic selection pressure to maintain plasmid-borne genes [6], [7]. Placing constructs in the bacterial chromosome can mitigate against these problems; however, chromosomal integrations can be highly variable in terms of integration efficiency and expression [8]. Moreover, there is limited information on the background and characterisation of integration sites. Identifying and characterising target loci for chromosomal expression is critical for reliable and rapid testing of genetic circuits. Since the expression of a gene is affected by its position in the chromosome [9], [10], we looked for loci containing non-essential genes with high expression under common laboratory conditions. We selected the flagellar regions as putative integration sites based on RNA-polymerase profiling (Figure 1 and Figure S1) and the observation that they are located in highly expressed genomic regions that are enriched for interactions with DNA-binding proteins (highly expressed extended protein occupancy domains, or heEPODs) [9]. To test the suitability of chromosomal loci for expression of genetic constructs, we chose to integrate a simple genetic construct consisting of the gene encoding the thermosensitive λ repressor cI857 and a constitutively active upstream promoter and ribosome binding site (RBS) elements from the Registry of Standard Biological Parts (Figure S2). The thermosensitive λ repressor carries a mutation that leads to denaturation of the repressor protein upon a temperature shift from 30°C to 42°C. At 30°C the repressor protein negatively regulates gene expression from the bacteriophage λ pR and pL promoters, but raising the temperature to 42 °C relieves repression allowing transcription from pR and pL [11]. We integrated this small genetic device into four promising sub-loci within flagellar region 3a. We characterised the sub-loci for efficiency of integration, level of transcription and expression. Finally, we propose *fliT* as standard site for the integration and characterisation of genetic devices.

## Results and Discussion

### Red recombinase and Gibson Assembly-based chromosomal integration strategy using pSB1K3(FRTK)

In order to simplify and standardise our integration method, we modified the well-characterised high copy number plasmid pSB1K3 by introducing the kanamycin-resistance gene flanked by FRT sites from the original pKD13 plasmid [12] (Materials and Methods and Figure S2). The resulting plasmid pSB1K3(FRTK) can easily accept virtually any genetic circuit for integration in the *E. coli* genome. We tested the new vector by synthesizing a 913 bp insert (Repr-ts-1) consisting of the thermosensitive λ repressor cI857, strong constitutive promoter (J23101), RBS (B0032) and double terminator (B0015) from the Registry of Standard Biological Parts. Isothermal Gibson Assembly was used to assemble Repr-ts-1 into pSB1K3(FRTK) to generate pSB1K3(FRTKr) (Figure S2). Repr-ts-1 was modified with flanking sequences derived from four genes in the flagellar region (see below) to produce a series of constructs ready for integration into the *E. coli* genome.

**Figure 2:**
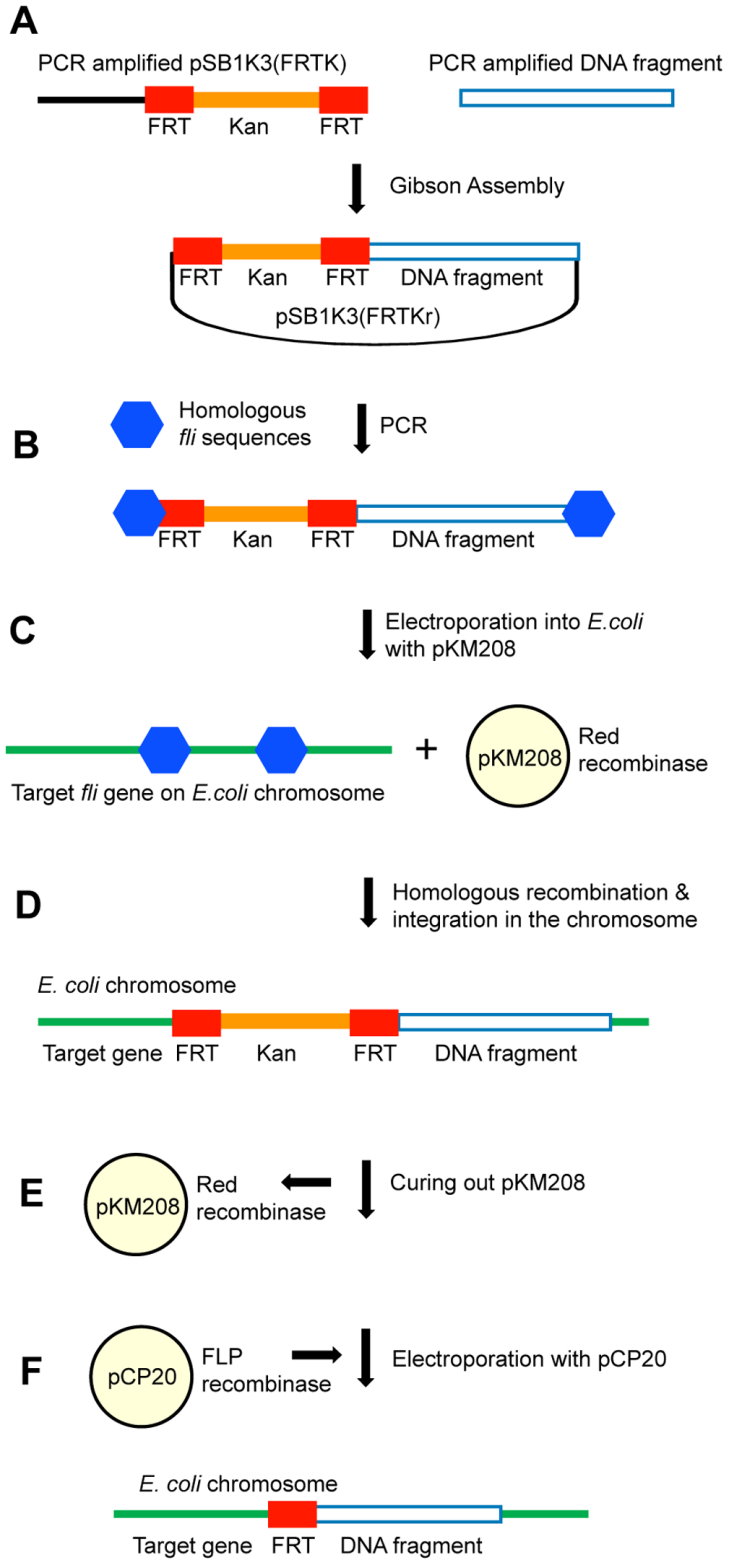
Chromosomal integration strategy. Figure depicts key steps of the DNA construct integration into the *E. coli* chromosome. (**A**) Gibson Assembly of the DNA fragment and pSB1K3(FRTK) plasmid backbone to make pSB1K3(FRTKr). (**B**) PCR amplification of the DNA fragment harboring the cI857 λ repressor construct, kanamycin and FRT sites flanked by sequences homologous to the target genes on the *E. coli* chromosome. (**C**) Electroporation of the DNA fragment into *E. coli* harboring plasmid pKM208 with Red recombinase system. (**D**) Red recombinase-induced recombination between homologous sequences integrates DNA fragment into the *E. coli* chromosome. (**E**) Temperature sensitive pKM208 is cured out from *E. coli* by growing at 42°C. (**F**) Plasmid pCP20 with FLP recombinase is electroporated into *E. coli* resulting in “flipping out” of the kanamycin from the chromosome.

Due to the low frequency of homologous recombination in *E. coli,* tools such as transposons, phages or more preferably phage-derived elements are used for engineering this bacterium [12]–[15]. We employed the pKM208 plasmid-borne Red recombinase system of the bacteriophage λ for integration of our DNA construct into the chromosome (Figure 2). The Red recombinase system includes three proteins, namely Gam, Bet and Exo. Gam inhibits the host RecBCD exonuclease V so that Bet and Exo can gain access to DNA ends to promote homologous recombination [12]. Red recombinase system in pKM208 is controlled by the lacZ promoter and regulated by the addition of Isopropyl β-D-1-thiogalactopyranoside (IPTG) to the growth media [16]. Our chromosomal integration protocol is simpler and more flexible than two other systems employing Red recombinase that have been developed recently. The first uses the yeast mitochondrial homing endonuclease I-SceI and requires a two step process for integration [15]. The second is based on knock-in/knock-out (KIKO) vectors and is restricted to integration at a selected few loci [14]. Other recent methods, such as ‘the one-step clonetegration’ [17] are capable of performing integration at similar if not faster rate but are limited in flexibility due to specific sequence requirements not present in subset of *E. coli* strains.

### Flagellar genes as target sites for chromosomal integration

The lack of well-characterized and reliable chromosomal integration sites is a major limitation for *E.coli* engineering. Sabri *et al* have recently reported the successful integration of foreign DNA into three *E. coli* chromosomal loci, namely the arsenite transporter *arsB*, the ribose transporter operon *rbsA-rbsR*, and the lactose catabolic enzyme-encoding *lacZ* [14]. Identification of other target sites for reliable chromosomal integration would significantly aid progress. We chose the *E. coli* K12 MG1655 flagellar gene region 3a (Figure 1) as a potentially suitable integration site for several reasons. First, the genes involved in flagella biogenesis and regulation are well-conserved in a wide variety of *E. coli* strains [10], [18]. Second, flagellar genes are considered to be non-essential for *E. coli* [13], thus we hypothesized that integration into these loci woud not be lethal for the cell. Third, the flagellar genes are well characterized and the interactions between them have been studied extensively [10], [18]. Fourth, suitable chromosomal integration sites during exponential growth should be located in chromosomal regions that show constitutive and high expression. RNA-seq and RNAP binding data obtained by Kahramanoglou *et al* [10] show that the flagellar genes are among the top 0.5% most highly expressed during *E. coli* exponential growth (Figure 1 and Figure S1). Fifth, the flagellar genes are found in heEPODs [9].

**Figure 3:**
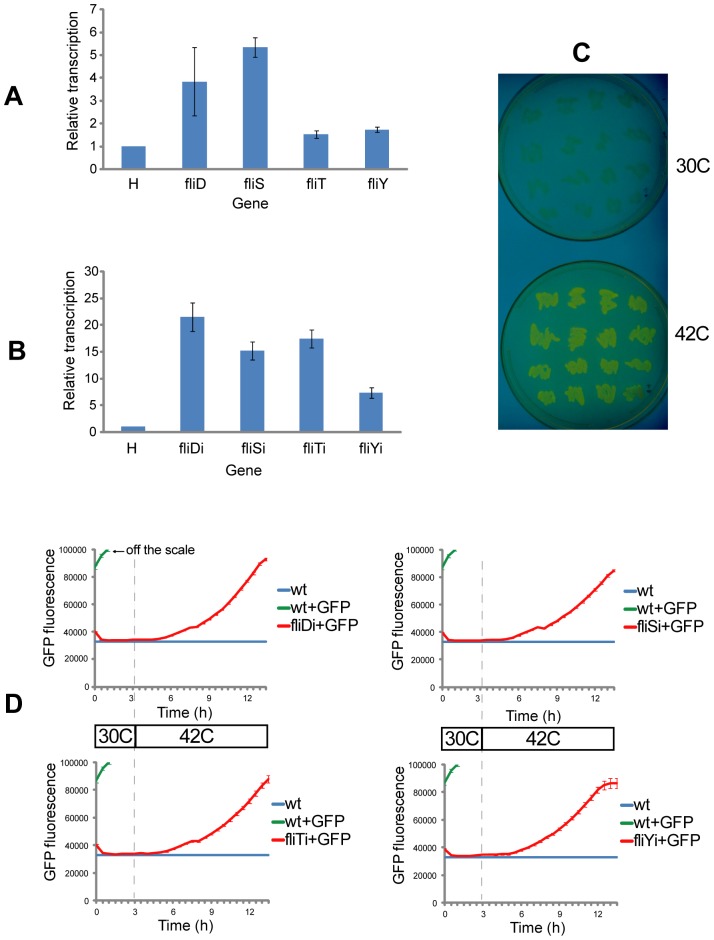
Expression of DNA integrated into flagellar region. (**A**) The relative expression levels of *fliD, fliS, fliT,* and *fliY* compared to the mean transcription of the two house-keeping genes *arcA* and *rpoD,* where each of the housekeeping genes was assayed in triplicate (H). Experiments were carried out in triplicate, error bars represent standard errors. (**B**) RT-PCR measured relative transcription of the thermosensitive λ repressor cI857 integrated into *fliD* (fliDi), *fliS* (fliSi), *fliY* (fliYi), and *fliT* (fliTi). These data show expression of the integrated DNA fragment compared to the mean transcription of the two house-keeping genes *arcA* and *rpoD,* where each of the housekeeping genes was assayed in triplicate (H). (**C**) Qualitative confirmation of the integration of Repr-ts-1 into the *E. coli* K12 MG1655 chromosome. At 30°C the λ repressor inhibits GFP expression and increasing the temperature to 42°C triggers GFP expression. (**D**) Quantitative confirmation of the integration of Repr-ts-1 into the *E. coli* strain K12 MG1655 chromosome by measuring GFP fluorescence over time with Fluostar Omega fluorimeter. Strains with integrations in *fliD* (fliDi), *fliS* (fliSi), *fliY* (fliYi), and *fliT* (fliTi) expressed GFP after the temperature shift to 42°C after 3 hours of growth (indicated by dashed line). In the control strain without repressor the GFP fluorescence signal saturated the fluorimeter detector (260000) after 5 hours of growth. Figure also shows that the integration of the repressor in the four flagellar genes had similar effect on the GFP expression. The starting absorbance (OD600) of the investigated strains was 0.05. Wt (wild type), wt+GFP (wild type transformed with the plasmid with the GFP located downstream of cI857– regulated pR promoter), rep+GFP (strain with the chromosomally- integrated cI857 transformed with the plasmid harboring pR controlled GFP).

We identified *fliD, fliS, fliT,* and *fliY* as putative chromosomal integration sites for our study. *fliD* encodes the cap that promotes filament-assembly [19], *fliS* and *fliT* encode specific export chaperones [18], [20], while *fliY* encodes cystine-binding periplasmic protein [21]. BLAST search revealed that all four selected flagellar genes are conserved among commonly-used *E. coli* strains, such as MG1655, W3110, DH10B and BL21-DE3. The location of the flagellar regions on the *E. coli* MG1655 chromosome and the location of the four target genes are shown in Figure 1 and Figure S1. These four genes are located in flagellar region 3a, which shows the highest levels of RNA polymerase binding (of the flagellar regions) at mid-exponential growth phase (Figure 1 and Figure S1). This indicates that this region of the chromosome is easily accessed by RNA polymerase, suggesting that DNA integrated into *fliD, fliS, fliT,* and *fliY* would be highly expressed. To confirm this, the relative expression of *fliD, fliS, fliT,* and *fliY* was measured by RT-PCR (Figure 3A). The expression at all four target genes showed higher expression (2–5 fold) compared to the mean expression of the housekeeping genes *arcA* and *rpoD* [22], [23]. Notably, the expression at *fliS* was 2–3 fold higher than at *fliD*, *fliT,* and *fliY* (Figure 3A).

### *fliT* supports high efficiency integration

**Figure 4:**
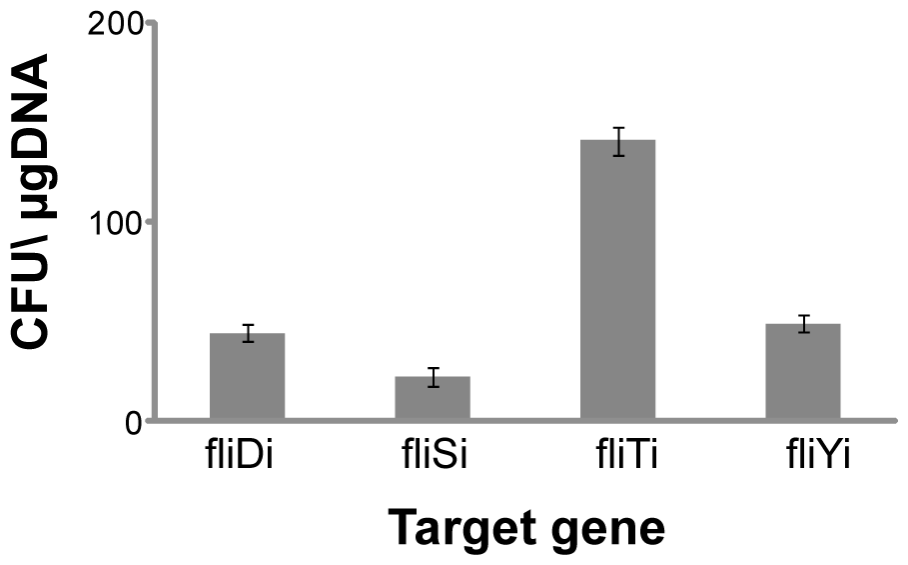
Variable efficiency of integration into flagellar genes. Figure shows the numbers of colonies per µg of electroporated DNA with the DNA fragment integrated in *fliD* (fliDi), *fliS* (fliSi), *fliY* (fliYi), and *fliT* (fliTi) of the *E. coli* flagellar region. The bars and errors represent averages and standard deviation calculated from three independent replicates.

We integrated our DNA construct Repr-ts-1 into each of four open reading frames of *fliD, fliS, fliT,* and *fliY*. Integration of Repr-ts-1 into the target loci and successful flipping out of the kanamycin resistance cassette was verified by diagnostic PCR and DNA sequencing (Figure S3). Interestingly, the integration efficiencies for Repr-ts-1 differed significantly for the four integration sites, despite their being located within the same flagellar region. The integration into *fliT* yielded three times the number of recombinants per µg DNA than integration into either *fliD* or *fliY*, and six times more recombinants than integration into *fliS* (Figure 4). The integration efficiencies do not appear to be operon-specific since *fliD, fliS*, and *fliT* are all part of the same operon, while *fliY* is part of a separate operon (Figure S1). The differences in the DNA integration efficiency might be attributed to the variable secondary structures in the sequences adjacent to target loci; further explanation would require investigation of the local 3D architecture of the *E. coli* chromosome. This suggests that the integration efficiency for any given locus must be determined empirically.

### Integration of DNA into flagellar genes reduces motility but does not inhibit cell growth

For the purpose of characterising genetic constructs, target loci for chromosomal integration should typically not negativelly impact growth rate. Essential genes should therefore be avoided [24]–[27]. Integration of the DNA fragment into all four flagellar genes yielded viable transformants whose growth was not inhibited relative to the wild type (Figure S4). The growth of the four engineered strains was impaired neither at the restrictive (37°C), nor permissive (30°C) temperatures for the cI857 repressor (Figure S4). Interestingly, strains with the integrated construct grew slightly better than the wild type strain (Figure S4 and Table S2). The engineered strains with Repr-ts-1 integrated in the flagellar genes had reduced motility when compared to the wild type (Figure S5). *fliD* and *fliS* mutants were most affected, while integration into *fliT* and *fliY* had only a minor effect on the motility (Figure S5). This could result in a lower metabolic burden to the cell due to the disruption of flagellar function. This experiment confirms that the flagellar region is suitable for the integration of DNA into the *E. coli* chromosome.

### Expression of cI857 from *fliD, fliS, fliT* and *fliY* ORFs

The transcription of cI857 integrated into *fliD* (fliDi), *fliS* (fliSi), *fliT* (fliTi) and *fliY* (fliYi) was measured by RT-PCR. Transcription was high in all four target sites (Figure 3B). The highest transcription levels were observed from *fliD* and *fliT*, at 20 and 18-fold that of the housekeeping genes (Figure 3B). Despite *fliT* showing the lowest relative transcription (Figure 3A), transcription of the integrated *cI857* was not significantly different than *cI857* integrated at *fliD* and *fliS* (Figure 3B). Expression from Repr-ts-1 was assessed qualitatively via the repressive activity of cI857. We constructed plasmid pSB1A1(GFP) harboring a GFP-expressing gene under the control of the cI857-repressible pR promoter and electroporated it into the *E. coli* strain containing chromosomally integrated Repr-ts-1. At 30°C GFP expression was inhibited but a temperature shift to 42°C triggered GFP expression (Figure 3C and D). The GFP expression was confirmed both qualitatively by visual observation (Figure 3C) and quantitatively by measuring GFP fluorescence over time (Figure 3D). These experiments confirmed that functional cI857 was expressed from the integrated constructs. Notwithstanding the differences in transcription levels of cI857 integrated at the four flagellar loci (Figure 3B), the level of derepression of GFP expression did not seem to be affected (Figure 3D).

## Conclusions

We set out to provide the Synthetic Biology community with well-characterised integration sites for expression of genetic circuits from the *E. coli* chromosome. The flagellar region appears to be a good target for the integration of genetic circuits into the *E. coli* chromosome. The integration of Repr-ts-1 into four genes of the flagellar region, namely *fliD*, *fliS*, *fliT* and *fliY* did not have any negative impact on growth. The mutants grew slightly better than the wild type strain, possibly as a result of the lower metabolic burden due to the disruption of the flagellar function. Notably, the four targeted genes of the flagellar region differed significantly in the efficiency of integration and level of transcription. Our results show that for the flagellar genes analysed, *fliT* is the best candidate site because of the high frequency of integration and high relative transcription. We also present a modified method for generating chromosomal integrations in *E. coli*. The method combines Isothermal Gibson Assembly of DNA fragments [28] with the Red system of the bacteriophage λ [12]. It is simpler and more flexible than the previously developed procedures of chromosomal integration employing Red recombinase, such as the I-SceI and KIKO vectors-based method. The transcriptional profile of flagellar genes [10] is better than that of phage attachment *att* sites utilized by the ‘clonetegration method’ [17]. Furthermore, whilst phage attachment sites [29] are often missing from some *E. coli* strains due to genotype changes, flagellar genes are well-conserved. BLAST search revealed that the four targeted flagellar genes are present in in all commonly-used *E. coli* strains, including MG1655, W3110, DH10B and BL21-DE3. Our identification and characterisation of flagellar integration sites will facilitate rational design of Synthetic Biology devices in *E. coli*.

## Materials and Methods

### Bacterial strains, plasmids, and growth conditions

Plasmids and bacterial strains used in this study are listed in Table 1. Luria-Bertani broth (LB) was used for cultivating *Escherichia coli* strains. When required, LB media was supplemented with ampicillin (100 µg/ml) or kanamycin (50 µg/ml). All plate cultures were cultivated for approximately 24 hours at either 30°C, 37°C or 42°C. Liquid cultures of *E. coli* were grown in LB and incubated at 200 r.p.m. on a rotatory shaker at 30°C, 37°C or 42°C, depending on the requirements.

**Table 1:** Bacterial strains and plasmids used in this study.

	Characteristics	Reference
**Strains**		
K12 MG1655	*E. coli* wild type	[36]
**Plasmids**		
pSB1K3	BioBrick assembly plasmid	Registry of St. Biological Parts
pSB1A1(GFP)	Amp^R^, λ promoter controlled GFP	This study
pKM208	Red recombinase controlled by lacZ	[16]
pCP20	FLP recombinase helper plasmid	[12]
pSB1K3(FRTK)	kanamycin FRT cassette in pSB1K3	This study
pSB1K3(FRTKr)	DNA construct (λ repressor) in pSB1K3(FRTK)	This study

### PCR amplification and DNA modification

Gel extraction and plasmid isolation were performed using standardized kits (Qiaquick Gel Extraction Kit and Qiaprep Spin Miniprep kit, respectively) from Qiagen according to the manufacturer’s instructions. Oligonucleotide primers used in this study were synthesized by IDT. PCR amplifications were routinely performed in 50 µl volumes using Phusion DNA polymerase (Thermo Scientific) or Dream Taq master mix kit (Thermo Scientific) according to the supplier’s instructions. Recombinant plasmids were confirmed by PCR amplification and sequencing.

### Gibson Isothermal Assembly of DNA fragments

A modified Gibson Isothermal Assembly method [28], [30] was used to clone target sequences into the plasmids. Briefly, 0.3 µl of the vector and 0.9 µl of the instert were added to 4 µl of the 1.33× Assembly Master Mix consisting of ISO Buffer, T5 exonuclease, Phusion polymerase, Taq ligase, and H_2_O to a final volume of 5.2 µl (http://www.srcf.ucam.org/~wac26/gibson/index.html). The assembly reaction was incubated at 50°C for 60 min and the whole volume was trasformed into competent *E. coli*.

### Construction of pSB1K3(FRT)

Plasmid pSB1K3(FRTK) was constructed by cloning the kanamycin resistance marker flanked by directly repeated FRT sites (originally from pKD13) [12] into pSB1K3. This was performed by joining DNA fragments with 40 to 60 bp overlapping sequences employing Isothermal Gibson Assembly. The first of the assembled fragments consisted of the kanamycin resistance gene flanked by FRT sites, while the second comprised the pSB1K3 backbone.

### Preparation and transformation of parental *E. coli*

Chemically competent *E. coli* have been prepared by a variation of the Hannah protocol using CCMB80 buffer [31], while electrocompetent *E. coli* were generated using the modified method of Miller and Nickoloff [32]. Briefly, parental *E. coli* strain K12 MG1655 was transformed with pKM208, recovered and selected on ampicillin plates at 30°C. 500 ml of LB with ampicillin was inoculated with overnight incubation (1∶100 dilution) of parental *E. coli* K12 MG1655 with pKM208, grown at 30°C to OD_600_ 0.2 prior to addition of IPTG (1 mM), then grown to a final OD_600_ of approximatelly 0.5. Cells were harvested by centrifugation, washed twice with 10% (v/v) glycerol and resuspended in a final volume of 100 µl of 10% glycerol per 100 ml of culture. 100 µl of the gel-purified PCR product (approximatelly 5 µg of DNA) was electroporated into 100 µl of prepared electro-competent cells carrying pKM208. Electrotransformation was performed on a Bio-Rad micropulser with the settings recommended for *E. coli* by the manufacturer.

### Selection of transformants and removal of antibiotic resistance gene

Transformants were selected on kanamycin plates at 37°C overnight. Kanamycin resistant colonies were incubated on LB plates without antibiotic at 42°C overnight to cure pKM208 plasmid and subsequently screened for kanamycin resistance and ampicillin sensitivity. Chromosomal integration was confirmed by diagnostic PCR with the flanking primers. Competent cells of the successful transformants were prepared by the method described above and transformed with the temperature sensitive plasmid pCP20 harboring FLP recombinase and selected on ampicillin plates at 30°C, overnight. Transformants were grown again on LB plates without antibiotic at 42°C overnight to cure pCP20 plasmid, tested for loss of kanamycin and ampicillin resistance and confirmed by PCR.

### Qualitative verification of the integrated DNA construct

To confirm the integration of cI857 λ repressor, constructed *E. coli* K12 MG1655 strain with integrated Repr-ts-1 was transformed with pSB1A1(GFP) which has GFP under the control of λ promoter (Figure 3C). Transformants were cultivated on plates at 30°C or 42°C.

### Plate reader measurement of the GFP fluorescence and absorbance

The overnight cultures were diluted to OD_600_ 0.05 in a total volume of 200 µl and transferred into flat-bottomed 96 well plates. Greiner BioOne black plates and Sterilin Sero-Well clear plates were used for the measurement of the GFP fluorescence and absorbance, respectively. The plates were incubated in a Fluostar Omega fluorimeter (BMG Labtech) at 37°C and 30°C for the measurement of absorbance and at 30°C and 42°C for the measurement of the GFP fluorescence with an automatically repeated protocol. Absorbance measurement was performed with the following parameters (600 nm absorbance filter, cycle time 30 min, number of cycles 48, double orbital shaking at 500 rpm). GFP fluorescence measurement was performed with the following parameters (excitation filter 485-12, emission filter EM520, gain 1400, cycle time 30 min, number of cycles: 6 at 30°C followed by 21 at 42°C, double orbital shaking at 200 rpm).

### RT-PCR expression analysis

Total RNA was isolated from strains grown into mid-exponential phase (OD_600_ = 0.7) using Isolate II RNA Mini Kit (Bioline) and purified from genomic DNA contamination with the help of a TURBO DNA-free Kit (Applied Biosystems). cDNA was prepared from 1 µg of total RNA employing SuperScript III Reverse Transcriptase (Invitrogen). Quantification of cDNA targets was performed with a QuantiTect SYBR Green PCR Kit (Qiagen). A 7500 Fast Real-Time PCR System (Applied Biosystems) was used to measure the expression levels of the target DNA sequences in MicroAmp Fast Optical 96-Well Reaction Plates (Applied Biosystems). Primers for RT-PCR were designed employing Primer3 Software. The relative expression levels were quantified with the help of REST9 Software (Qiagen) employing Pfaffl method [33]. The experiments were carried out in triplicate and the means and standard errors were calculated.

### Motility assay

Motility agar plates were prepared by pouring 100 ml of the motility agar (10 g tryptone, 5 g NaCl, 0.25% Bacto-Agar (Difco)) in the 13 cm plate and let to set overnight. Motility plates were pre-warmed at 37°C before the experiment. Overnight cultures of the tested bacterial strains were normalized to an absorbance (OD600) of 1.0 prior to spotting 2 µl of the cultures into the middle of the motility plates. Plates were grown for 4–6 hours at 37°C.

### Sequence analyses and databases

DNA sequences were compared with the help of the National Center for Biotechnology Information (NCBI) website (http://ncbi.nlm.nih.gov) using the BLASTN [34], TBLASTX algorithms and position-specific iterated BLAST (PSI-BLAST) [35]. The annotated *E. coli* K-12 genome was obtained from the *E. coli* K-12 project website (http://www.xbase.ac.uk/genome/escherichia-coli-str-k-12-substr-mg1655). The DNA construct was designed from standard biological parts catalogued in the Registry of Standard Biological Parts (http://parts.igem.org/Main_Page?title=Main_Page). DNA sequencing was performed by Source Bioscience (Cambridge, UK).

## Supporting Information

Figure S1 **Expression of the** ***E. coli*** **flagellum regions 2 and 3.** The *E. coli* K12 MG1655 genome showing RNA polymerase (RNA-POL) binding (green peaks; ChIP-seq data from cells at mid-exponential growth phase [10]). Figure was generated by uploading the RNA-Pol binding data (from Kahramanoglou *et al*) [10] to the UCSC microbial genome browser for *E. coli* K12 MG1655 (http://microbes.ucsc.edu/cgi-bin/hgGateway?db=eschColi_K12). Two regions (1962580–1978197 bp and 1999585–2023678 bp) are expanded to show the positions of the highly expressed genes of the *E. coli* flagellum regions 2 and 3. Integration target sites (*fliD, S, T, Y*) are located in the two highly expressed operons of the *E. coli* K12 MG1655 flagellar gene region 3a.
(TIF)

Figure S2 **pSB1K3(FRTKr) plasmid map.** Figure shows the main features of the constructed plasmid pSB1K3(FRTKr). FRT (directly repeated FRT sites); Kan (kanamycin), Prom (promoter); RBS (ribosomal binding site); cI857 (thermosensitive λ repressor); Term (terminator).
(TIF)

Figure S3 **PCR verification of the chromosomal integration.** Figure shows the result of confirmation of the integration of the synthetic DNA construct in *fliD* (fliDi), *fliS* (fliSi), *fliY* (fliYi), and *fliT* (fliTi) of the *E. coli* strain K12 MG1655 chromosome using flanking primers. Wt (wild type), k (integrated DNA fragment with kanamycin resistance), −k (integrated DNA fragment from which the kanamycin resistance was flipped out). HyperLadder 1kb (Bioline) has been used as the molecular weight marker.
(TIF)

Figure S4 **Growth rate of strains with integrated DNA.** Growth curves of the wild type *E. coli* K12 MG1655 (wt) and strains harboring synthetic DNA fragment integrated in *fliD* (fliDi), *fliS* (fliSi), *fliY* (fliYi), and *fliT* (fliTi). Values are the means calculated from three independent experiments. Raw plate reader data, means and standard deviations are in the Table S1.
(TIF)

Figure S5 **Motility Assay.** The engineered strains harboring integrated thermosensitive repressor in the flagellar genes had reduced motility when compared to the wild type (wt). Overnight cultures of the tested bacterial strains were normalized to an absorbance (OD600) of 1.0 prior to spotting 2 µl of the cultures into the middle of the motility plates. Picture was taken after 5 hours of incubation at 37°C.
(TIF)

Table S1 **Primers used in this study.**
(DOC)

Table S2 **Growth rates of** ***Escherichia coli*** **strains with integrated DNA.**
(DOT)

## References

[1] Juhas M, Davenport PW, Brown JR, Yarkoni O, Ajioka JW (2013) Meeting report: The Cambridge BioDesign TechEvent - Synthetic Biology, a new “Age of Wonder”? Biotechnol J.

[2] Chen X, Zhou L, Tian K, Kumar A, Singh S, et al (2013) Metabolic engineering of Escherichia coli: a sustainable industrial platform for bio-based chemical production. Biotechnol Adv 31: 1200–1223. 23473968 10.1016/j.biotechadv.2013.02.009

[3] Park SJ, Lee TW, Lim SC, Kim TW, Lee H, et al (2012) Biosynthesis of polyhydroxyalkanoates containing 2-hydroxybutyrate from unrelated carbon source by metabolically engineered Escherichia coli. Appl Microbiol Biotechnol 93: 273–283. 21842437 10.1007/s00253-011-3530-x

[4] Yim H, Haselbeck R, Niu W, Pujol-Baxley C, Burgard A, et al (2011) Metabolic engineering of Escherichia coli for direct production of 1,4-butanediol. Nat Chem Biol 7: 445–452. 21602812 10.1038/nchembio.580

[5] Ajikumar PK, Xiao WH, Tyo KE, Wang Y, Simeon F, et al (2010) Isoprenoid pathway optimization for Taxol precursor overproduction in Escherichia coli. Science 330: 70–74. 20929806 10.1126/science.1191652 PMC3034138

[6] Cunningham DS, Koepsel RR, Ataai MM, Domach MM (2009) Factors affecting plasmid production in Escherichia coli from a resource allocation standpoint. Microb Cell Fact 8: 27. 19463175 10.1186/1475-2859-8-27 PMC2702362

[7] Marcellin E, Chen WY, Nielsen LK (2010) Understanding plasmid effect on hyaluronic acid molecular weight produced by Streptococcus equi subsp. zooepidemicus. Metab Eng 12: 62–69. 19782148 10.1016/j.ymben.2009.09.001

[8] Das B, Bischerour J, Val ME, Barre FX (2010) Molecular keys of the tropism of integration of the cholera toxin phage. Proc Natl Acad Sci U S A 107: 4377–4382. 20133778 10.1073/pnas.0910212107 PMC2840090

[9] Vora T, Hottes AK, Tavazoie S (2009) Protein occupancy landscape of a bacterial genome. Mol Cell 35: 247–253. 19647521 10.1016/j.molcel.2009.06.035 PMC2763621

[10] Kahramanoglou C, Seshasayee AS, Prieto AI, Ibberson D, Schmidt S, et al (2011) Direct and indirect effects of H-NS and Fis on global gene expression control in Escherichia coli. Nucleic Acids Res 39: 2073–2091. 21097887 10.1093/nar/gkq934 PMC3064808

[11] Mieschendahl M, Müller-Hill B (1985) F'-coded, temperature-sensitive lambda cI857 repressor gene for easy construction and regulation of lambda promoter-dependent expression systems. J Bacteriol 164: 1366–1369. 2999084 10.1128/jb.164.3.1366-1369.1985 PMC219341

[12] Datsenko KA, Wanner BL (2000) One-step inactivation of chromosomal genes in Escherichia coli K-12 using PCR products. Proc Natl Acad Sci U S A 97: 6640–6645. 10829079 10.1073/pnas.120163297 PMC18686

[13] Baba T, Ara T, Hasegawa M, Takai Y, Okumura Y, et al (2006) Construction of Escherichia coli K-12 in-frame, single-gene knockout mutants: the Keio collection. Mol Syst Biol 2: 2006.0008. 10.1038/msb4100050 PMC1681482 16738554

[14] Sabri S, Steen JA, Bongers M, Nielsen LK, Vickers CE (2013) Knock-in/Knock-out (KIKO) vectors for rapid integration of large DNA sequences, including whole metabolic pathways, onto the Escherichia coli chromosome at well-characterised loci. Microb Cell Fact 12: 60. 23799955 10.1186/1475-2859-12-60 PMC3706339

[15] Ublinskaya AA, Samsonov VV, Mashko SV, Stoynova NV (2012) A PCR-free cloning method for the targeted φ80 Int-mediated integration of any long DNA fragment, bracketed with meganuclease recognition sites, into the Escherichia coli chromosome. J Microbiol Methods 89: 167–173. 22484061 10.1016/j.mimet.2012.03.013

[16] Murphy KC, Campellone KG (2003) Lambda Red-mediated recombinogenic engineering of enterohemorrhagic and enteropathogenic E. coli. BMC Mol Biol 4: 11. 14672541 10.1186/1471-2199-4-11 PMC317293

[17] St-Pierre F, Cui L, Priest DG, Endy D, Dodd IB, et al (2013) One-step cloning and chromosomal integration of DNA. ACS Synth Biol 2: 537–541. 24050148 10.1021/sb400021j

[18] Fraser GM, Bennett JC, Hughes C (1999) Substrate-specific binding of hook-associated proteins by FlgN and FliT, putative chaperones for flagellum assembly. Mol Microbiol 32: 569–580. 10320579 10.1046/j.1365-2958.1999.01372.x

[19] Ikeda T, Oosawa K, Hotani H (1996) Self-assembly of the filament capping protein, FliD, of bacterial flagella into an annular structure. J Mol Biol 259: 679–686. 8683574 10.1006/jmbi.1996.0349

[20] Ozin AJ, Claret L, Auvray F, Hughes C (2003) The FliS chaperone selectively binds the disordered flagellin C-terminal D0 domain central to polymerisation. FEMS Microbiol Lett 219: 219–224. 12620624 10.1016/S0378-1097(02)01208-9

[21] Keseler IM, Collado-Vides J, Gama-Castro S, Ingraham J, Paley S, et al (2005) EcoCyc: a comprehensive database resource for Escherichia coli. Nucleic Acids Res 33: D334–337. 15608210 10.1093/nar/gki108 PMC540062

[22] Minty JJ, Lesnefsky AA, Lin F, Chen Y, Zaroff TA, et al (2011) Evolution combined with genomic study elucidates genetic bases of isobutanol tolerance in Escherichia coli. Microb Cell Fact 10: 18. 21435272 10.1186/1475-2859-10-18 PMC3071312

[23] Jandu N, Ho NK, Donato KA, Karmali MA, Mascarenhas M, et al (2009) Enterohemorrhagic Escherichia coli O157:H7 gene expression profiling in response to growth in the presence of host epithelia. PLoS One 4: e4889. 19293938 10.1371/journal.pone.0004889 PMC2654852

[24] Juhas M, Eberl L, Church GM (2012) Essential genes as antimicrobial targets and cornerstones of synthetic biology. Trends Biotechnol 30: 601–607. 22951051 10.1016/j.tibtech.2012.08.002

[25] Juhas M, Eberl L, Glass JI (2011) Essence of life: essential genes of minimal genomes. Trends Cell Biol 21: 562–568. 21889892 10.1016/j.tcb.2011.07.005

[26] Juhas M, Stark M, von Mering C, Lumjiaktase P, Crook DW, et al (2012) High confidence prediction of essential genes in Burkholderia cenocepacia. PLoS One 7: e40064. 22768221 10.1371/journal.pone.0040064 PMC3386938

[27] Juhas M, Reuß D, Zhu B, Commichau FM (2014) Bacillus subtilis and Escherichia coli essential genes and minimal cell factories after one decade of genome engineering. Microbiology. 10.1099/mic.0.079376-0 25092907

[28] Gibson D, Young L, Chuang R, Venter J, Hutchison Cr, et al (2009) Enzymatic assembly of DNA molecules up to several hundred kilobases. Nat Methods 6: 343–345. 19363495 10.1038/nmeth.1318

[29] Haldimann A, Wanner BL (2001) Conditional-replication, integration, excision, and retrieval plasmid-host systems for gene structure-function studies of bacteria. J Bacteriol 183: 6384–6393. 11591683 10.1128/JB.183.21.6384-6393.2001 PMC100134

[30] Merryman C, Gibson DG (2012) Methods and applications for assembling large DNA constructs. Metab Eng 14: 196–204. 22629570 10.1016/j.ymben.2012.02.005

[31] Hanahan D, Jessee J, Bloom FR (1991) Plasmid transformation of Escherichia coli and other bacteria. Methods Enzymol 204: 63–113. 1943786 10.1016/0076-6879(91)04006-a

[32] Miller EM, Nickoloff JA (1995) Escherichia coli electrotransformation. Methods Mol Biol 47: 105–113. 7550724 10.1385/0-89603-310-4:105

[33] Pfaffl MW, Horgan GW, Dempfle L (2002) Relative expression software tool (REST) for group-wise comparison and statistical analysis of relative expression results in real-time PCR. Nucleic Acids Res 30: e36. 11972351 10.1093/nar/30.9.e36 PMC113859

[34] Altschul SF, Gish W, Miller W, Myers EW, Lipman DJ (1990) Basic local alignment search tool. J Mol Biol 215: 403–410. 2231712 10.1016/S0022-2836(05)80360-2

[35] Altschul SF, Madden TL, Schaffer AA, Zhang J, Zhang Z, et al (1997) Gapped BLAST and PSI-BLAST: a new generation of protein database search programs. Nucleic Acids Res 25: 3389–3402. 9254694 10.1093/nar/25.17.3389 PMC146917

[36] Hayashi K, Morooka N, Yamamoto Y, Fujita K, Isono K, et al (2006) Highly accurate genome sequences of Escherichia coli K-12 strains MG1655 and W3110. Mol Syst Biol 2: 2006.0007. 10.1038/msb4100049 PMC1681481 16738553

