# Supplementary figures and images for: Escherichia coli Flagellar Genes as Target Sites for Integration and Expression of Genetic Circuits
Source: PLoS One. 2014 Oct 28;9(10):e111451. doi: 10.1371/journal.pone.0111451 (PMC4211737; doi:10.1371/journal.pone.0111451)

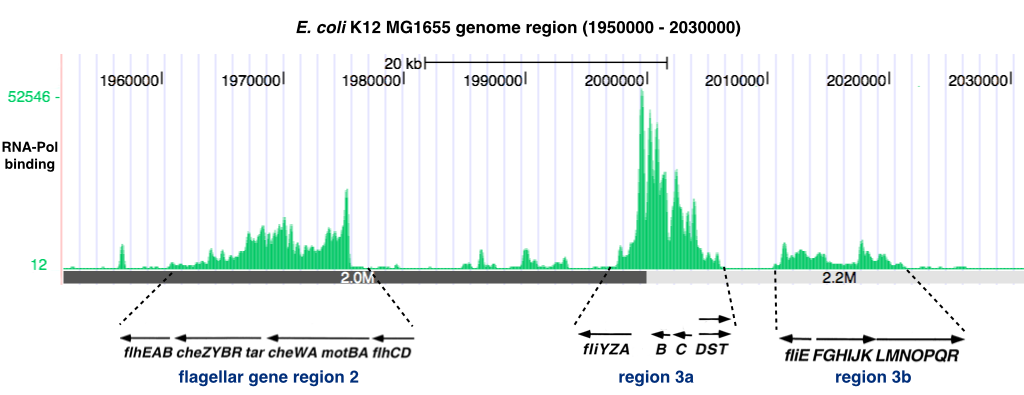

Supplement: Figure S1 — Expression of the E. coli flagellum regions 2 and 3. The E. coli K12 MG1655 genome showing RNA polymerase (RNA-POL) binding (green peaks; ChIP-seq data from cells at mid-exponential growth phase [10]). Figure was generated by uploading the RNA-Pol binding data (from Kahramanoglou et al) [10] to the UCSC microbial genome browser for E. coli K12 MG1655 (http://microbes.ucsc.edu/cgi-bin/hgGateway?db=eschColi_K12). Two regions (1962580–1978197 bp and 1999585–2023678 bp) are expanded to show the positions of the highly expressed genes of the E. coli flagellum regions 2 and 3. Integration target sites (fliD, S, T, Y) are located in the two highly expressed operons of the E. coli K12 MG1655 flagellar gene region 3a. (TIF) [file pone.0111451.s001.tif]

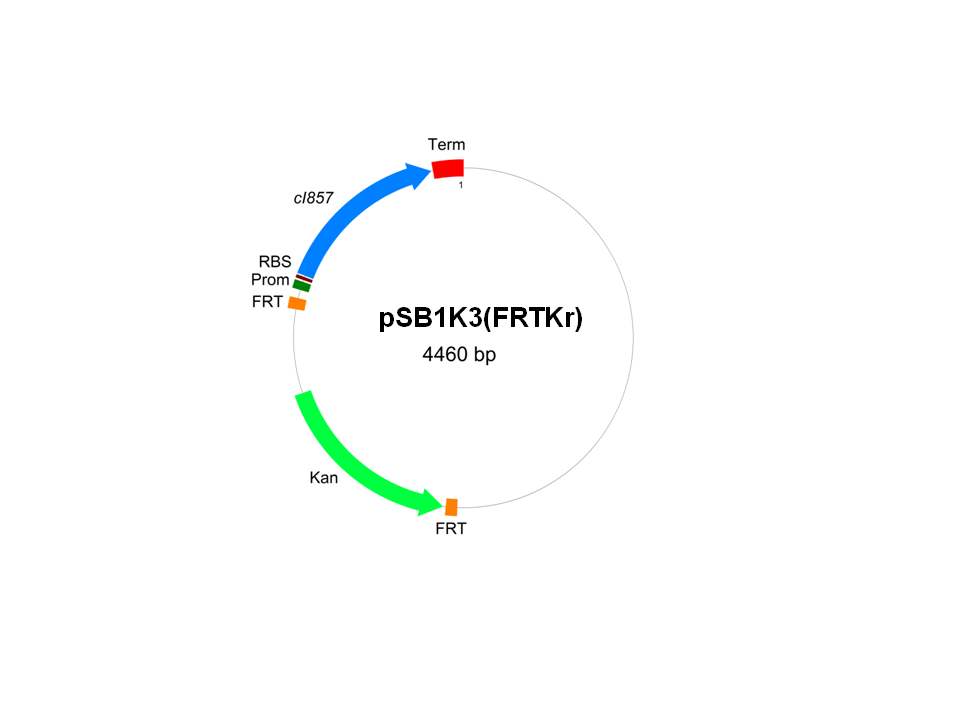

Supplement: Figure S2 — pSB1K3(FRTKr) plasmid map. Figure shows the main features of the constructed plasmid pSB1K3(FRTKr). FRT (directly repeated FRT sites); Kan (kanamycin), Prom (promoter); RBS (ribosomal binding site); cI857 (thermosensitive λ repressor); Term (terminator). (TIF) [file pone.0111451.s002.tif]

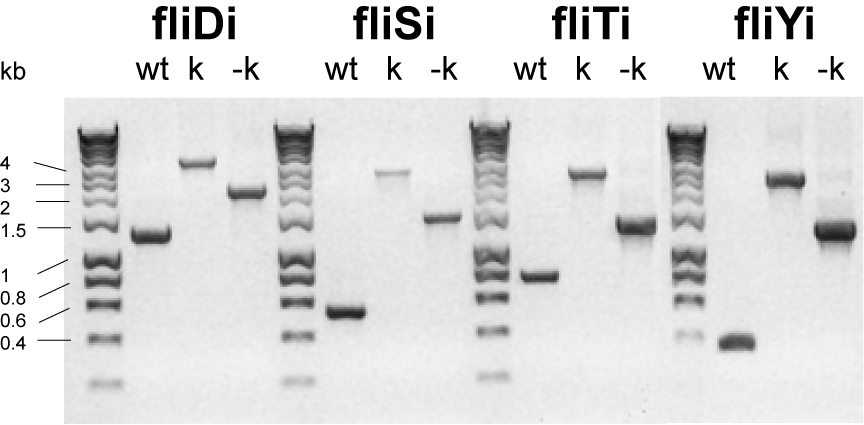

Supplement: Figure S3 — PCR verification of the chromosomal integration. Figure shows the result of confirmation of the integration of the synthetic DNA construct in fliD (fliDi), fliS (fliSi), fliY (fliYi), and fliT (fliTi) of the E. coli strain K12 MG1655 chromosome using flanking primers. Wt (wild type), k (integrated DNA fragment with kanamycin resistance), −k (integrated DNA fragment from which the kanamycin resistance was flipped out). HyperLadder 1kb (Bioline) has been used as the molecular weight marker. (TIF) [file pone.0111451.s003.tif]

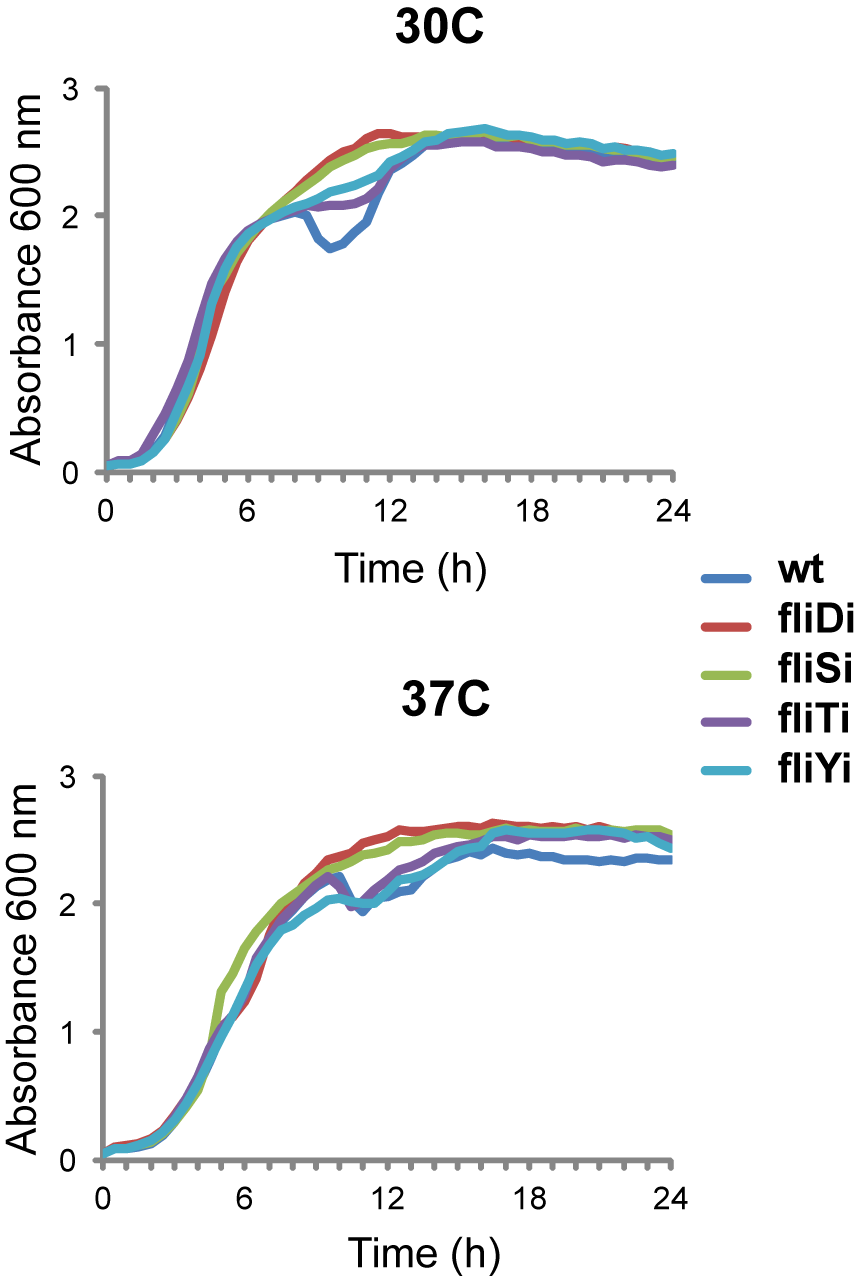

Supplement: Figure S4 — Growth rate of strains with integrated DNA. Growth curves of the wild type E. coli K12 MG1655 (wt) and strains harboring synthetic DNA fragment integrated in fliD (fliDi), fliS (fliSi), fliY (fliYi), and fliT (fliTi). Values are the means calculated from three independent experiments. Raw plate reader data, means and standard deviations are in the Table S1. (TIF) [file pone.0111451.s004.tif]

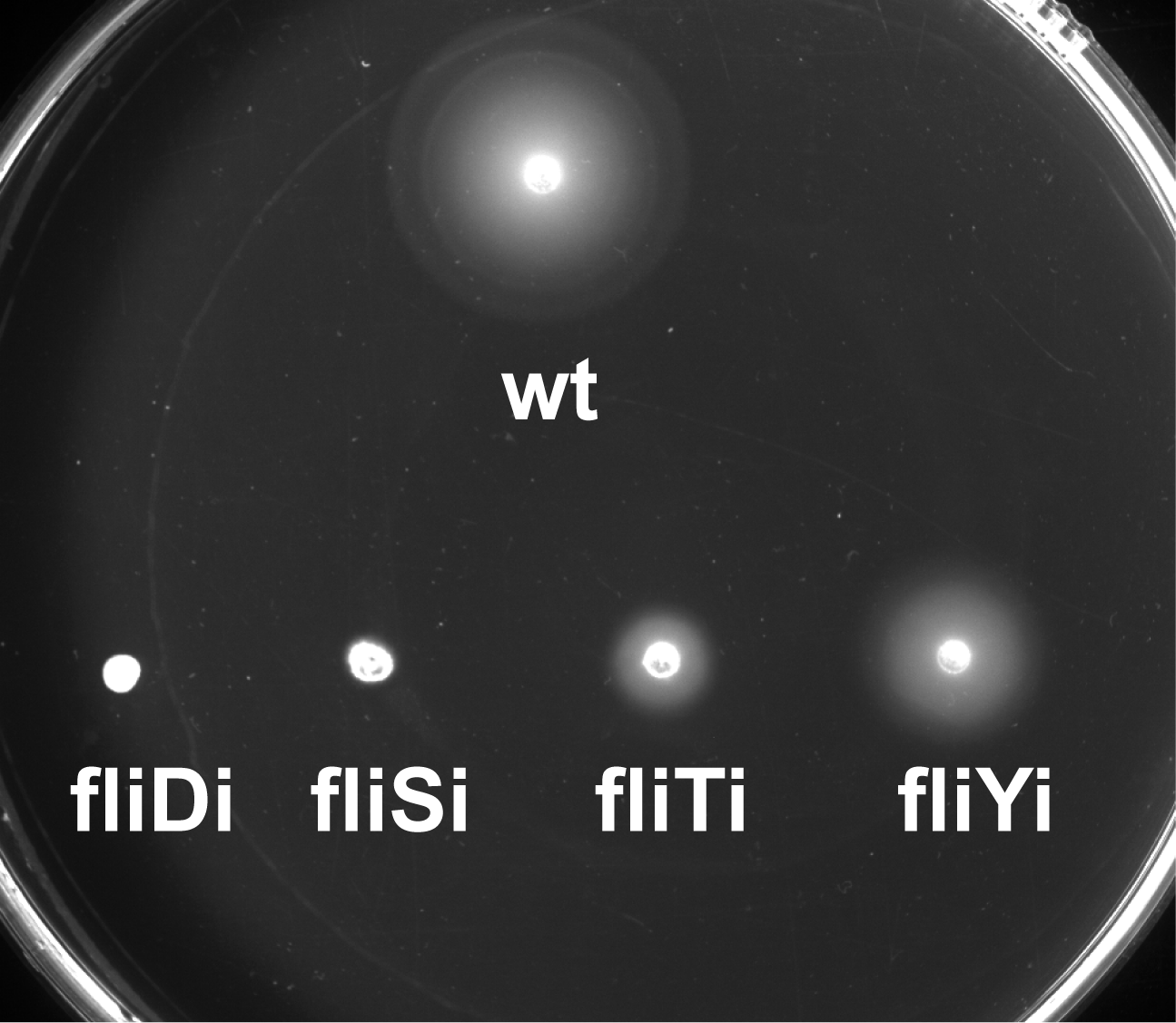

Supplement: Figure S5 — Motility Assay. The engineered strains harboring integrated thermosensitive repressor in the flagellar genes had reduced motility when compared to the wild type (wt). Overnight cultures of the tested bacterial strains were normalized to an absorbance (OD600) of 1.0 prior to spotting 2 µl of the cultures into the middle of the motility plates. Picture was taken after 5 hours of incubation at 37°C. (TIF) [file pone.0111451.s005.tif]
