# Supplementary material for: Escherichia coli Flagellar Genes as Target Sites for Integration and Expression of Genetic Circuits
Source: PLoS One. 2014 Oct 28;9(10):e111451. doi: 10.1371/journal.pone.0111451 (PMC4211737; doi:10.1371/journal.pone.0111451)
Supplement: Table S1 — Primers used in this study. (DOC) [file pone.0111451.s006.doc]

**Supporting Table 1**. Primers used in this study.

| **Primer (Sequence 5’→ 3’)** |
| --- |
| fliDrepF: *fliD* integration primer forward |
| GTGAATATCGTCTGTCAGTCACATCAAATGACACCGGCCTTGATAATGCGATGACACTCTCGGTCAGTATCACGAGGCAGAATTTCAGAT |
| fliDrepR: *fliD* integration primer reverse |
| TTTGGTCAGGCTGCTGAAGGTATCTATTAGCGAGTTGTAGGCATTCACCCAGTCTTTAATCGCCGTTCGGTTTTAAAGAAAAAGGGCAGG |
| fliSrepF: *fliS* integration primer forward |
| TTATGCAGGACAACAATCAGCAAGGCAAAGGCGTCTCTTTGTCAAAAGCGATCAACATCATTGAGAGTATCACGAGGCAGAATTTCAGAT |
| fliSrepR: *fliS* integration primer reverse |
| AATATTGCGCATTAATGCTTCCACTTCTTCGACTGCGGAGACATCGTTGCGTAAATTGGCTTCGGTTTTAAAGAAAAAGGGCAGG |
| fliTrepF: *fliT* integration primer forward |
| CTGATTCTCGACAACGAAAGCAAGGTAAAGCAGTTATTACAGATTCGGATGGATGAACTGGCGAAAGTATCACGAGGCAGAATTTCAGAT |
| fliTrepR: *fliT* integration primer reverse |
| TGAAAACATTGTAACAGCGTGGGATTACGCATTCTTCGACTCCATTCAAGGGGAACATTAGAACGGTTTTAAAGAAAAAGGGCAGG |
| fliYrepF: *fliY* integration primer forward |
| TATGCTGGCGTCGCTGGACTCTAAACGTATTGATGTGGTGATTAATCAGGTCACCATTTCTGATGAGTATCACGAGGCAGAATTTCAGAT |
| fliYrepR: *fliY* integration primer reverse |
| TGTGTGTTGTTGTGATTTTCTTATTATGCACGCTGAAAACGCGTAAATAAAAAAGGCGCTAGTGAAACGGTTTTAAAGAAAAAGGGCAGG |
| pSB1K3(FRTK)I1: primer 1 for Gibson assembly of FRTKanamycin cassette and pSB1K3 |
| CGGGTGGGCCTTTCTGCGTTTATATTTACAGCTAGCTCAGTCCTAG |
| pSB1K3(FRTK)I2: primer 2 for Gibson assembly of FRTKanamycin cassette and pSB1K3 |
| CCTTGCCCTTTTTTGCCGGATATAAACGCAGAAAGGCCCA |
| pSB1K3(FRTK)V1: primer 3 for Gibson assembly of FRTKanamycin cassette and pSB1K3 |
| TGGGCCTTTCTGCGTTTATATCCGGCAAAAAAGGGCAAGG |
| pSB1K3(FRTK)V2: primer 4 for Gibson assembly of FRTKanamycin cassette and pSB1K3 |
| CTAGGACTGAGCTAGCTGTAAATATAAACGCAGAAAGGCCCACCCG |
| pSB1K3(FRTKr)I1: primer 1 for Gibson assembly of cI857 construct and pSB1K3(FRTK) |
| CTTTCGCTAAGGATGATTTCTGGAAGATTGCAGCATTACACGTCTTGAGCG |
| pSB1K3(FRTKr)I2: primer 2 for Gibson assembly of cI857 construct and pSB1K3(FRTK) |
| CATAATACCTAGGACTGAGCTAGCTGTAAATGAGAATTAATTCCGGGGATCCGTCGAC |
| pSB1K3(FRTKr)V1: primer 3 for Gibson assembly of cI857 construct and pSB1K3(FRTK) |
| GTCGACGGATCCCCGGAATTAATTCTCATTTACAGCTAGCTCAGTCCTAGGTATTATG |
| pSB1K3(FRTKr)V2: primer 4 for Gibson assembly of cI857 construct and pSB1K3(FRTK) |
| CGCTCAAGACGTGTAATGCTGCAATCTTCCAGAAATCATCCTTAGCGAAAG |
