# Supplementary material for: Escherichia coli Flagellar Genes as Target Sites for Integration and Expression of Genetic Circuits
Source: PLoS One. 2014 Oct 28;9(10):e111451. doi: 10.1371/journal.pone.0111451 (PMC4211737; doi:10.1371/journal.pone.0111451)
Supplement: Table S2 — Growth rates of Escherichia coli strains with integrated DNA. (DOT) [file pone.0111451.s007.dot]

**Table S2. Growth rates of *Escherichia coli* strains with integrated DNA.**

|  | wild type 37°C | | | | |
| --- | --- | --- | --- | --- | --- |
| Time (h) | 1 | 2 | 3 | Average | StDev |
| 0 | 0.05 | 0.05 | 0.05 | 0.05 | 0 |
|  | 0.0811 | 0.0811 | 0.0821 | 0.081433 | 0.000577 |
| 1 | 0.0813 | 0.0804 | 0.0824 | 0.081367 | 0.001002 |
|  | 0.0973 | 0.0966 | 0.0996 | 0.097833 | 0.00157 |
| 2 | 0.1257 | 0.1278 | 0.1323 | 0.1286 | 0.003372 |
|  | 0.1846 | 0.1952 | 0.2005 | 0.193433 | 0.008096 |
| 3 | 0.2684 | 0.3016 | 0.309 | 0.293 | 0.021623 |
|  | 0.3865 | 0.4339 | 0.451 | 0.4238 | 0.033415 |
| 4 | 0.5383 | 0.5812 | 0.5966 | 0.572033 | 0.030212 |
|  | 0.7218 | 0.7733 | 0.7854 | 0.760167 | 0.033773 |
| 5 | 0.946 | 0.9777 | 0.9996 | 0.974433 | 0.026949 |
|  | 1.0493 | 1.1055 | 1.2151 | 1.1233 | 0.084321 |
| 6 | 1.2024 | 1.3033 | 1.4333 | 1.313 | 0.115755 |
|  | 1.4328 | 1.5138 | 1.6621 | 1.536233 | 0.116284 |
| 7 | 1.6216 | 1.7307 | 1.8455 | 1.7326 | 0.111962 |
|  | 1.7736 | 1.865 | 1.9632 | 1.867267 | 0.09482 |
| 8 | 1.8872 | 1.9514 | 2.035 | 1.957867 | 0.074112 |
|  | 2.0234 | 2.0499 | 2.0897 | 2.054333 | 0.033372 |
| 9 | 2.1235 | 2.1309 | 2.1341 | 2.1295 | 0.005437 |
|  | 2.2015 | 2.1919 | 2.1725 | 2.188633 | 0.014773 |
| 10 | 2.2598 | 2.226 | 2.1497 | 2.211833 | 0.056401 |
|  | 1.9651 | 2.0309 | 2.0853 | 2.0271 | 0.06019 |
| 11 | 1.8344 | 1.8253 | 2.1426 | 1.9341 | 0.180624 |
|  | 1.9739 | 2.0573 | 2.1142 | 2.048467 | 0.070566 |
| 12 | 2.0399 | 1.9974 | 2.1143 | 2.050533 | 0.059171 |
|  | 2.0675 | 2.1022 | 2.1278 | 2.099167 | 0.030264 |
| 13 | 1.9658 | 2.1487 | 2.2059 | 2.1068 | 0.125414 |
|  | 2.0585 | 2.2883 | 2.2987 | 2.215167 | 0.135777 |
| 14 | 2.0526 | 2.4046 | 2.3771 | 2.2781 | 0.195772 |
|  | 2.1696 | 2.4516 | 2.4255 | 2.3489 | 0.155826 |
| 15 | 2.1271 | 2.503 | 2.4803 | 2.370133 | 0.210779 |
|  | 2.2211 | 2.4933 | 2.5245 | 2.412967 | 0.166892 |
| 16 | 2.1258 | 2.4971 | 2.5294 | 2.3841 | 0.224277 |
|  | 2.2032 | 2.5478 | 2.5683 | 2.439767 | 0.205129 |
| 17 | 2.0991 | 2.5267 | 2.5761 | 2.400633 | 0.262301 |
|  | 2.1053 | 2.4704 | 2.5568 | 2.3775 | 0.239658 |
| 18 | 2.1491 | 2.4679 | 2.5618 | 2.392933 | 0.216322 |
|  | 2.1207 | 2.4629 | 2.5433 | 2.375633 | 0.224409 |
| 19 | 2.0556 | 2.5052 | 2.5636 | 2.3748 | 0.277973 |
|  | 1.9869 | 2.4818 | 2.5485 | 2.339067 | 0.306803 |
| 20 | 1.9801 | 2.4696 | 2.5689 | 2.339533 | 0.315213 |
|  | 1.9816 | 2.4877 | 2.5633 | 2.3442 | 0.316288 |
| 21 | 1.9753 | 2.4888 | 2.5453 | 2.336467 | 0.314053 |
|  | 1.9719 | 2.4862 | 2.5565 | 2.3382 | 0.319167 |
| 22 | 1.9643 | 2.4891 | 2.5486 | 2.334 | 0.321549 |
|  | 1.9699 | 2.5045 | 2.5812 | 2.351867 | 0.333008 |
| 23 | 1.9674 | 2.5039 | 2.5804 | 2.350567 | 0.334029 |
|  | 1.9567 | 2.497 | 2.5781 | 2.343933 | 0.337797 |
| 24 | 1.9478 | 2.506 | 2.5756 | 2.343133 | 0.344133 |

|  | fliDi 37°C | | | | |
| --- | --- | --- | --- | --- | --- |
| Time (h) | 1 | 2 | 3 | Average | StDev |
| 0 | 0.05 | 0.05 | 0.05 | 0.05 | 0 |
|  | 0.0942 | 0.0963 | 0.0947 | 0.095067 | 0.001097 |
| 1 | 0.11 | 0.1153 | 0.1038 | 0.1097 | 0.005756 |
|  | 0.1197 | 0.1283 | 0.1247 | 0.124233 | 0.004319 |
| 2 | 0.1547 | 0.1723 | 0.1676 | 0.164867 | 0.009113 |
|  | 0.217 | 0.2454 | 0.2422 | 0.234867 | 0.015555 |
| 3 | 0.3144 | 0.3564 | 0.3546 | 0.3418 | 0.023746 |
|  | 0.4442 | 0.4847 | 0.4832 | 0.4707 | 0.022962 |
| 4 | 0.5826 | 0.6575 | 0.659 | 0.633033 | 0.043683 |
|  | 0.7876 | 0.8756 | 0.8795 | 0.847567 | 0.051969 |
| 5 | 1.0022 | 1.0605 | 1.0517 | 1.038133 | 0.031429 |
|  | 1.0985 | 1.1372 | 1.1287 | 1.121467 | 0.020339 |
| 6 | 1.235 | 1.233 | 1.2297 | 1.232567 | 0.002676 |
|  | 1.5707 | 1.3464 | 1.3498 | 1.4223 | 0.128529 |
| 7 | 1.8698 | 1.6803 | 1.7161 | 1.7554 | 0.100677 |
|  | 2.0552 | 1.9068 | 1.9528 | 1.9716 | 0.075965 |
| 8 | 2.157 | 1.9159 | 1.9795 | 2.017467 | 0.124954 |
|  | 2.2441 | 2.1005 | 2.1284 | 2.157667 | 0.076142 |
| 9 | 2.3048 | 2.2024 | 2.2141 | 2.240433 | 0.056049 |
|  | 2.3843 | 2.3194 | 2.3369 | 2.346867 | 0.033578 |
| 10 | 2.4135 | 2.3215 | 2.3725 | 2.369167 | 0.04609 |
|  | 2.4541 | 2.3655 | 2.3756 | 2.3984 | 0.048501 |
| 11 | 2.5044 | 2.4618 | 2.4716 | 2.479267 | 0.022311 |
|  | 2.5195 | 2.4984 | 2.4969 | 2.504933 | 0.012637 |
| 12 | 2.528 | 2.5142 | 2.5244 | 2.5222 | 0.007158 |
|  | 2.5976 | 2.5576 | 2.5649 | 2.573367 | 0.021302 |
| 13 | 2.5705 | 2.5576 | 2.5725 | 2.566867 | 0.008087 |
|  | 2.5553 | 2.5625 | 2.5637 | 2.5605 | 0.004543 |
| 14 | 2.5635 | 2.5974 | 2.5818 | 2.5809 | 0.016968 |
|  | 2.5674 | 2.5905 | 2.5994 | 2.585767 | 0.016517 |
| 15 | 2.5687 | 2.6201 | 2.6327 | 2.607167 | 0.033904 |
|  | 2.5733 | 2.6144 | 2.6177 | 2.6018 | 0.024737 |
| 16 | 2.5712 | 2.605 | 2.6131 | 2.596433 | 0.022225 |
|  | 2.6129 | 2.6189 | 2.6449 | 2.625567 | 0.01701 |
| 17 | 2.6133 | 2.6087 | 2.6268 | 2.616267 | 0.009408 |
|  | 2.614 | 2.594 | 2.6043 | 2.6041 | 0.010001 |
| 18 | 2.6082 | 2.5808 | 2.6125 | 2.6005 | 0.017196 |
|  | 2.5888 | 2.58 | 2.599 | 2.589267 | 0.009509 |
| 19 | 2.6109 | 2.5785 | 2.6215 | 2.603633 | 0.022402 |
|  | 2.6107 | 2.5679 | 2.599 | 2.592533 | 0.022121 |
| 20 | 2.5907 | 2.5679 | 2.6438 | 2.6008 | 0.038945 |
|  | 2.567 | 2.5418 | 2.6177 | 2.5755 | 0.038657 |
| 21 | 2.6193 | 2.575 | 2.6182 | 2.604167 | 0.025265 |
|  | 2.5776 | 2.5685 | 2.5956 | 2.580567 | 0.013791 |
| 22 | 2.5561 | 2.5426 | 2.5329 | 2.543867 | 0.011652 |
|  | 2.5594 | 2.5629 | 2.5718 | 2.5647 | 0.006393 |
| 23 | 2.5474 | 2.5688 | 2.5691 | 2.561767 | 0.012443 |
|  | 2.5287 | 2.5447 | 2.5525 | 2.541967 | 0.012133 |
| 24 | 2.4668 | 2.5334 | 2.5326 | 2.510933 | 0.038223 |

|  | fliSi 37°C | | | | |
| --- | --- | --- | --- | --- | --- |
| Time (h) | 1 | 2 | 3 | Average | StDev |
| 0 | 0.05 | 0.05 | 0.05 | 0.05 | 0 |
|  | 0.0812 | 0.0872 | 0.0868 | 0.085067 | 0.003355 |
| 1 | 0.0829 | 0.0877 | 0.0906 | 0.087067 | 0.003889 |
|  | 0.1004 | 0.1067 | 0.1104 | 0.105833 | 0.005056 |
| 2 | 0.1307 | 0.1427 | 0.1494 | 0.140933 | 0.009474 |
|  | 0.1833 | 0.1999 | 0.2133 | 0.198833 | 0.015028 |
| 3 | 0.2629 | 0.2925 | 0.3154 | 0.290267 | 0.026321 |
|  | 0.3748 | 0.4157 | 0.4311 | 0.4072 | 0.029097 |
| 4 | 0.4975 | 0.545 | 0.5743 | 0.538933 | 0.038758 |
|  | 0.7591 | 0.7683 | 0.87 | 0.799133 | 0.061544 |
| 5 | 1.2388 | 1.3069 | 1.3749 | 1.306867 | 0.06805 |
|  | 1.4171 | 1.4585 | 1.5104 | 1.462 | 0.046748 |
| 6 | 1.6127 | 1.6386 | 1.6886 | 1.646633 | 0.038582 |
|  | 1.7614 | 1.7851 | 1.8206 | 1.789033 | 0.029795 |
| 7 | 1.8754 | 1.9031 | 1.9324 | 1.903633 | 0.028504 |
|  | 1.9819 | 2.0057 | 2.0218 | 2.003133 | 0.020073 |
| 8 | 2.0548 | 2.0757 | 2.0971 | 2.075867 | 0.02115 |
|  | 2.1139 | 2.1416 | 2.1684 | 2.1413 | 0.027251 |
| 9 | 2.1723 | 2.1975 | 2.2125 | 2.1941 | 0.020315 |
|  | 2.2441 | 2.2645 | 2.2736 | 2.260733 | 0.015106 |
| 10 | 2.2784 | 2.2944 | 2.3083 | 2.2937 | 0.014962 |
|  | 2.3015 | 2.3278 | 2.3563 | 2.328533 | 0.027407 |
| 11 | 2.3571 | 2.3774 | 2.4007 | 2.3784 | 0.021817 |
|  | 2.3831 | 2.3932 | 2.4154 | 2.397233 | 0.016523 |
| 12 | 2.391 | 2.427 | 2.4518 | 2.423267 | 0.030571 |
|  | 2.4617 | 2.4912 | 2.4965 | 2.483133 | 0.01875 |
| 13 | 2.471 | 2.4838 | 2.5158 | 2.4902 | 0.023076 |
|  | 2.5003 | 2.5009 | 2.5121 | 2.504433 | 0.006646 |
| 14 | 2.5394 | 2.5354 | 2.5405 | 2.538433 | 0.002684 |
|  | 2.5359 | 2.5571 | 2.5703 | 2.554433 | 0.017354 |
| 15 | 2.5538 | 2.5502 | 2.5627 | 2.555567 | 0.006435 |
|  | 2.5261 | 2.5497 | 2.562 | 2.545933 | 0.018244 |
| 16 | 2.5285 | 2.5437 | 2.528 | 2.5334 | 0.008924 |
|  | 2.5812 | 2.5603 | 2.5471 | 2.562867 | 0.017194 |
| 17 | 2.5825 | 2.6163 | 2.5807 | 2.593167 | 0.020054 |
|  | 2.5612 | 2.5543 | 2.5681 | 2.5612 | 0.0069 |
| 18 | 2.5692 | 2.5804 | 2.6027 | 2.5841 | 0.017054 |
|  | 2.5559 | 2.5642 | 2.5665 | 2.5622 | 0.005576 |
| 19 | 2.5751 | 2.5727 | 2.5653 | 2.571033 | 0.005108 |
|  | 2.5544 | 2.5791 | 2.5598 | 2.564433 | 0.012986 |
| 20 | 2.5806 | 2.5646 | 2.614 | 2.5864 | 0.025206 |
|  | 2.5497 | 2.5508 | 2.6147 | 2.571733 | 0.037214 |
| 21 | 2.5231 | 2.5491 | 2.6226 | 2.564933 | 0.051605 |
|  | 2.541 | 2.5739 | 2.6255 | 2.580133 | 0.042593 |
| 22 | 2.5473 | 2.5397 | 2.6045 | 2.563833 | 0.035423 |
|  | 2.5438 | 2.5721 | 2.6396 | 2.585167 | 0.049219 |
| 23 | 2.5494 | 2.5592 | 2.648 | 2.585533 | 0.054319 |
|  | 2.5578 | 2.5456 | 2.6285 | 2.5773 | 0.044758 |
| 24 | 2.4948 | 2.5342 | 2.586 | 2.538333 | 0.04574 |

|  | fliTi 37°C | | | | |
| --- | --- | --- | --- | --- | --- |
| Time (h) | 1 | 2 | 3 | Average | StDev |
| 0 | 0.05 | 0.05 | 0.05 | 0.05 | 0 |
|  | 0.0905 | 0.0882 | 0.086 | 0.088233 | 0.00225 |
| 1 | 0.09 | 0.0912 | 0.0878 | 0.089667 | 0.001724 |
|  | 0.1115 | 0.1107 | 0.1078 | 0.11 | 0.001947 |
| 2 | 0.147 | 0.1497 | 0.1451 | 0.147267 | 0.002312 |
|  | 0.2097 | 0.2249 | 0.2166 | 0.217067 | 0.007611 |
| 3 | 0.3076 | 0.3519 | 0.3374 | 0.3323 | 0.022586 |
|  | 0.4495 | 0.4907 | 0.4777 | 0.472633 | 0.021062 |
| 4 | 0.5853 | 0.6803 | 0.6628 | 0.6428 | 0.050559 |
|  | 0.809 | 0.9069 | 0.8962 | 0.8707 | 0.053701 |
| 5 | 1.0052 | 1.042 | 1.0318 | 1.026333 | 0.018999 |
|  | 1.1168 | 1.1423 | 1.1414 | 1.1335 | 0.01447 |
| 6 | 1.206 | 1.414 | 1.2379 | 1.285967 | 0.112021 |
|  | 1.4878 | 1.6301 | 1.5873 | 1.5684 | 0.073008 |
| 7 | 1.6188 | 1.8352 | 1.7219 | 1.7253 | 0.10824 |
|  | 1.8284 | 1.9405 | 1.871 | 1.879967 | 0.056585 |
| 8 | 1.864 | 2.0779 | 1.96 | 1.9673 | 0.107137 |
|  | 1.9438 | 2.1417 | 2.0712 | 2.052233 | 0.100304 |
| 9 | 2.0456 | 2.2319 | 2.1842 | 2.1539 | 0.096775 |
|  | 2.1265 | 2.2907 | 2.2411 | 2.219433 | 0.084217 |
| 10 | 1.9892 | 2.2323 | 2.1875 | 2.136333 | 0.129375 |
|  | 1.9166 | 2.0057 | 2.0268 | 1.983033 | 0.058492 |
| 11 | 1.8562 | 2.1009 | 2.1051 | 2.020733 | 0.142506 |
|  | 2.0062 | 2.16 | 2.1663 | 2.110833 | 0.09067 |
| 12 | 2.1128 | 2.2127 | 2.2473 | 2.190933 | 0.069842 |
|  | 2.2218 | 2.2684 | 2.3146 | 2.268267 | 0.0464 |
| 13 | 2.2761 | 2.2779 | 2.3362 | 2.296733 | 0.034191 |
|  | 2.3375 | 2.291 | 2.3735 | 2.334 | 0.041361 |
| 14 | 2.4283 | 2.3257 | 2.4207 | 2.391567 | 0.057169 |
|  | 2.4653 | 2.3519 | 2.4631 | 2.426767 | 0.064846 |
| 15 | 2.4917 | 2.3844 | 2.4836 | 2.453233 | 0.059749 |
|  | 2.5046 | 2.3989 | 2.4893 | 2.464267 | 0.057124 |
| 16 | 2.5319 | 2.4108 | 2.5178 | 2.486833 | 0.066223 |
|  | 2.5884 | 2.435 | 2.5385 | 2.520633 | 0.078245 |
| 17 | 2.5893 | 2.4432 | 2.5604 | 2.530967 | 0.07737 |
|  | 2.5617 | 2.4185 | 2.5409 | 2.507033 | 0.077374 |
| 18 | 2.6583 | 2.4215 | 2.5498 | 2.5432 | 0.118538 |
|  | 2.6356 | 2.4116 | 2.5494 | 2.5322 | 0.112986 |
| 19 | 2.6191 | 2.4261 | 2.5537 | 2.532967 | 0.098156 |
|  | 2.6275 | 2.416 | 2.5501 | 2.5312 | 0.107009 |
| 20 | 2.6359 | 2.4191 | 2.5546 | 2.536533 | 0.109523 |
|  | 2.6339 | 2.4101 | 2.552 | 2.532 | 0.113233 |
| 21 | 2.6188 | 2.4114 | 2.5568 | 2.529 | 0.106458 |
|  | 2.6316 | 2.4068 | 2.5655 | 2.534633 | 0.115535 |
| 22 | 2.6067 | 2.3853 | 2.5396 | 2.510533 | 0.113526 |
|  | 2.6362 | 2.404 | 2.5779 | 2.539367 | 0.120801 |
| 23 | 2.6346 | 2.3916 | 2.551 | 2.525733 | 0.123455 |
|  | 2.6194 | 2.4008 | 2.557 | 2.525733 | 0.112604 |
| 24 | 2.6001 | 2.3801 | 2.5349 | 2.505033 | 0.113 |

|  | fliYi 37°C | | | | |
| --- | --- | --- | --- | --- | --- |
| Time (h) | 1 | 2 | 3 | Average | StDev |
| 0 | 0.05 | 0.05 | 0.05 | 0.05 | 0 |
|  | 0.0869 | 0.0966 | 0.0886 | 0.0907 | 0.00518 |
| 1 | 0.0863 | 0.0979 | 0.0877 | 0.090633 | 0.006332 |
|  | 0.1033 | 0.1216 | 0.1091 | 0.111333 | 0.009352 |
| 2 | 0.1315 | 0.1619 | 0.1483 | 0.147233 | 0.015228 |
|  | 0.1819 | 0.2344 | 0.2284 | 0.2149 | 0.028736 |
| 3 | 0.2452 | 0.3238 | 0.3366 | 0.301867 | 0.04949 |
|  | 0.3512 | 0.4664 | 0.4851 | 0.434233 | 0.072514 |
| 4 | 0.4958 | 0.6371 | 0.6585 | 0.597133 | 0.088407 |
|  | 0.6951 | 0.7974 | 0.8362 | 0.776233 | 0.072893 |
| 5 | 0.9045 | 0.9641 | 0.9938 | 0.954133 | 0.045477 |
|  | 0.9954 | 1.1708 | 1.2282 | 1.131467 | 0.121282 |
| 6 | 1.1633 | 1.3902 | 1.4197 | 1.3244 | 0.140294 |
|  | 1.4119 | 1.5866 | 1.5785 | 1.525667 | 0.098608 |
| 7 | 1.5912 | 1.7408 | 1.6845 | 1.672167 | 0.075559 |
|  | 1.7381 | 1.863 | 1.7773 | 1.7928 | 0.063876 |
| 8 | 1.8138 | 1.8959 | 1.813 | 1.8409 | 0.047633 |
|  | 1.9061 | 1.9496 | 1.8946 | 1.916767 | 0.02901 |
| 9 | 1.9832 | 1.9797 | 1.9494 | 1.970767 | 0.018587 |
|  | 2.0656 | 2.0011 | 2.009 | 2.025233 | 0.035181 |
| 10 | 2.0903 | 1.987 | 2.0349 | 2.0374 | 0.051695 |
|  | 2.1101 | 1.9179 | 2.0189 | 2.015633 | 0.096142 |
| 11 | 2.026 | 1.9291 | 2.0673 | 2.007467 | 0.07094 |
|  | 1.8785 | 2.0575 | 2.0768 | 2.004267 | 0.109344 |
| 12 | 1.8701 | 2.2125 | 2.1947 | 2.092433 | 0.192752 |
|  | 1.927 | 2.3319 | 2.3033 | 2.1874 | 0.225966 |
| 13 | 1.8752 | 2.3652 | 2.3562 | 2.198867 | 0.28034 |
|  | 1.9065 | 2.3763 | 2.3841 | 2.2223 | 0.273519 |
| 14 | 2.0015 | 2.4248 | 2.4209 | 2.2824 | 0.243274 |
|  | 2.1306 | 2.4567 | 2.4533 | 2.346867 | 0.1873 |
| 15 | 2.2689 | 2.4673 | 2.4913 | 2.409167 | 0.122066 |
|  | 2.3142 | 2.488 | 2.506 | 2.436067 | 0.105923 |
| 16 | 2.3586 | 2.4867 | 2.4911 | 2.445467 | 0.075261 |
|  | 2.5395 | 2.5581 | 2.5464 | 2.548 | 0.009403 |
| 17 | 2.6002 | 2.5832 | 2.5636 | 2.582333 | 0.018315 |
|  | 2.5533 | 2.5811 | 2.5586 | 2.564333 | 0.01476 |
| 18 | 2.4936 | 2.5986 | 2.5775 | 2.556567 | 0.055542 |
|  | 2.4754 | 2.6133 | 2.5648 | 2.551167 | 0.069954 |
| 19 | 2.4795 | 2.616 | 2.5753 | 2.556933 | 0.070079 |
|  | 2.4763 | 2.6092 | 2.5883 | 2.557933 | 0.071465 |
| 20 | 2.4862 | 2.6336 | 2.5952 | 2.571667 | 0.076466 |
|  | 2.5037 | 2.6244 | 2.6098 | 2.5793 | 0.065877 |
| 21 | 2.5009 | 2.6095 | 2.6192 | 2.576533 | 0.06568 |
|  | 2.4801 | 2.602 | 2.6142 | 2.565433 | 0.074152 |
| 22 | 2.481 | 2.5816 | 2.598 | 2.553533 | 0.063349 |
|  | 2.3874 | 2.5571 | 2.5989 | 2.514467 | 0.11201 |
| 23 | 2.3816 | 2.582 | 2.6202 | 2.527933 | 0.12816 |
|  | 2.2956 | 2.5563 | 2.5814 | 2.477767 | 0.158259 |
| 24 | 2.1927 | 2.5363 | 2.5785 | 2.435833 | 0.211614 |

|  | wild type 30°C | | | | |
| --- | --- | --- | --- | --- | --- |
| Time (h) | 1 | 2 | 3 | Average | StDev |
| 0 | 0.05 | 0.05 | 0.05 | 0.05 | 0 |
|  | 0.0657 | 0.0653 | 0.0663 | 0.065767 | 0.000503 |
| 1 | 0.0659 | 0.0661 | 0.0675 | 0.0665 | 0.000872 |
|  | 0.087 | 0.0881 | 0.0921 | 0.089067 | 0.002684 |
| 2 | 0.1603 | 0.1585 | 0.1744 | 0.1644 | 0.008707 |
|  | 0.2777 | 0.2723 | 0.2996 | 0.2832 | 0.014457 |
| 3 | 0.4928 | 0.49 | 0.5229 | 0.5019 | 0.01824 |
|  | 0.7064 | 0.7032 | 0.7452 | 0.718267 | 0.02338 |
| 4 | 0.8998 | 0.925 | 0.9833 | 0.936033 | 0.042829 |
|  | 1.2824 | 1.2722 | 1.3252 | 1.293267 | 0.028121 |
| 5 | 1.5591 | 1.5001 | 1.562 | 1.5404 | 0.034931 |
|  | 1.7255 | 1.6927 | 1.7777 | 1.731967 | 0.042867 |
| 6 | 1.8222 | 1.839 | 1.9179 | 1.8597 | 0.051098 |
|  | 1.8699 | 1.9371 | 1.9907 | 1.932567 | 0.060527 |
| 7 | 1.8796 | 2.0098 | 2.0366 | 1.975333 | 0.083983 |
|  | 1.9217 | 2.0328 | 2.0667 | 2.007067 | 0.075848 |
| 8 | 1.9504 | 2.0699 | 2.0965 | 2.038933 | 0.077817 |
|  | 1.9709 | 2.0183 | 2.0413 | 2.010167 | 0.035898 |
| 9 | 1.8534 | 1.7805 | 1.8317 | 1.821867 | 0.037432 |
|  | 1.7046 | 1.7322 | 1.8026 | 1.746467 | 0.050534 |
| 10 | 1.7092 | 1.7945 | 1.8553 | 1.786333 | 0.073392 |
|  | 1.8089 | 1.9027 | 1.9246 | 1.878733 | 0.061461 |
| 11 | 1.9016 | 1.954 | 1.989 | 1.9482 | 0.043988 |
|  | 2.0813 | 2.2028 | 2.236 | 2.173367 | 0.081442 |
| 12 | 2.3207 | 2.3795 | 2.3745 | 2.358233 | 0.032601 |
|  | 2.4073 | 2.4093 | 2.417 | 2.4112 | 0.005122 |
| 13 | 2.4799 | 2.4706 | 2.4741 | 2.474867 | 0.004697 |
|  | 2.5713 | 2.5371 | 2.5629 | 2.5571 | 0.017822 |
| 14 | 2.5821 | 2.5675 | 2.5657 | 2.571767 | 0.008994 |
|  | 2.611 | 2.5848 | 2.606 | 2.6006 | 0.01391 |
| 15 | 2.6483 | 2.6206 | 2.5982 | 2.622367 | 0.025097 |
|  | 2.6281 | 2.627 | 2.6066 | 2.620567 | 0.012108 |
| 16 | 2.6252 | 2.6264 | 2.6241 | 2.625233 | 0.00115 |
|  | 2.6238 | 2.6031 | 2.5707 | 2.5992 | 0.026764 |
| 17 | 2.6098 | 2.6033 | 2.5967 | 2.603267 | 0.00655 |
|  | 2.5978 | 2.6361 | 2.589 | 2.607633 | 0.025042 |
| 18 | 2.5642 | 2.578 | 2.5258 | 2.556 | 0.027049 |
|  | 2.5424 | 2.5386 | 2.5061 | 2.529033 | 0.019952 |
| 19 | 2.5242 | 2.5342 | 2.5088 | 2.5224 | 0.012795 |
|  | 2.4996 | 2.5216 | 2.4849 | 2.502033 | 0.018471 |
| 20 | 2.5047 | 2.4992 | 2.4615 | 2.488467 | 0.023515 |
|  | 2.5043 | 2.4891 | 2.4629 | 2.485433 | 0.020942 |
| 21 | 2.4841 | 2.4968 | 2.4302 | 2.470367 | 0.03536 |
|  | 2.4839 | 2.4832 | 2.4115 | 2.459533 | 0.0416 |
| 22 | 2.4885 | 2.439 | 2.4107 | 2.446067 | 0.039378 |
|  | 2.4956 | 2.4561 | 2.3782 | 2.4433 | 0.059738 |
| 23 | 2.4906 | 2.4057 | 2.3873 | 2.427867 | 0.055102 |
|  | 2.4618 | 2.3691 | 2.3947 | 2.408533 | 0.047873 |
| 24 | 2.4795 | 2.3868 | 2.3997 | 2.422 | 0.050212 |

|  | fliDi 30°C | | | | |
| --- | --- | --- | --- | --- | --- |
| Time (h) | 1 | 2 | 3 | Average | StDev |
| 0 | 0.05 | 0.05 | 0.05 | 0.05 | 0 |
|  | 0.0686 | 0.0703 | 0.0694 | 0.069433 | 0.00085 |
| 1 | 0.0763 | 0.0776 | 0.0758 | 0.076567 | 0.000929 |
|  | 0.0925 | 0.0971 | 0.0988 | 0.096133 | 0.003259 |
| 2 | 0.1452 | 0.1493 | 0.154 | 0.1495 | 0.004403 |
|  | 0.2543 | 0.2522 | 0.26 | 0.2555 | 0.004036 |
| 3 | 0.3845 | 0.4027 | 0.4121 | 0.399767 | 0.014032 |
|  | 0.5673 | 0.5868 | 0.6056 | 0.586567 | 0.019151 |
| 4 | 0.7847 | 0.8006 | 0.8239 | 0.803067 | 0.019716 |
|  | 1.1268 | 1.0015 | 1.0839 | 1.070733 | 0.063679 |
| 5 | 1.4533 | 1.2925 | 1.4881 | 1.4113 | 0.104345 |
|  | 1.6443 | 1.6194 | 1.6712 | 1.644967 | 0.025906 |
| 6 | 1.7885 | 1.7976 | 1.8375 | 1.807867 | 0.026063 |
|  | 1.8824 | 1.9092 | 1.9513 | 1.9143 | 0.034732 |
| 7 | 1.9896 | 2.003 | 2.0569 | 2.0165 | 0.035623 |
|  | 2.068 | 2.0919 | 2.1585 | 2.106133 | 0.046899 |
| 8 | 2.1604 | 2.174 | 2.2527 | 2.1957 | 0.04983 |
|  | 2.2302 | 2.2723 | 2.3313 | 2.277933 | 0.050785 |
| 9 | 2.308 | 2.3423 | 2.4137 | 2.354667 | 0.053924 |
|  | 2.4134 | 2.4312 | 2.48 | 2.441533 | 0.034481 |
| 10 | 2.4758 | 2.4866 | 2.5504 | 2.504267 | 0.040316 |
|  | 2.5217 | 2.5178 | 2.5639 | 2.534467 | 0.025564 |
| 11 | 2.5849 | 2.5733 | 2.6675 | 2.608567 | 0.051366 |
|  | 2.6437 | 2.5987 | 2.6899 | 2.6441 | 0.045601 |
| 12 | 2.655 | 2.6181 | 2.6542 | 2.642433 | 0.021077 |
|  | 2.6189 | 2.5955 | 2.6436 | 2.619333 | 0.024053 |
| 13 | 2.6115 | 2.6236 | 2.6164 | 2.617167 | 0.006086 |
|  | 2.5939 | 2.6329 | 2.6512 | 2.626 | 0.029267 |
| 14 | 2.585 | 2.5918 | 2.6365 | 2.604433 | 0.027978 |
|  | 2.5935 | 2.6184 | 2.6463 | 2.6194 | 0.026414 |
| 15 | 2.5995 | 2.6137 | 2.64 | 2.617733 | 0.020549 |
|  | 2.6075 | 2.6109 | 2.663 | 2.627133 | 0.031108 |
| 16 | 2.617 | 2.6222 | 2.6568 | 2.632 | 0.021634 |
|  | 2.6138 | 2.6021 | 2.6519 | 2.6226 | 0.02604 |
| 17 | 2.5943 | 2.6144 | 2.6359 | 2.614867 | 0.020804 |
|  | 2.6163 | 2.5858 | 2.6348 | 2.6123 | 0.024744 |
| 18 | 2.5869 | 2.5711 | 2.6271 | 2.595033 | 0.028872 |
|  | 2.5757 | 2.5717 | 2.6124 | 2.5866 | 0.022433 |
| 19 | 2.6008 | 2.587 | 2.6094 | 2.599067 | 0.0113 |
|  | 2.6005 | 2.5439 | 2.5713 | 2.5719 | 0.028305 |
| 20 | 2.6218 | 2.5299 | 2.593 | 2.581567 | 0.047005 |
|  | 2.6062 | 2.5192 | 2.5814 | 2.568933 | 0.04482 |
| 21 | 2.5526 | 2.4926 | 2.5555 | 2.533567 | 0.035508 |
|  | 2.5629 | 2.4905 | 2.5666 | 2.54 | 0.042908 |
| 22 | 2.5761 | 2.4643 | 2.5338 | 2.524733 | 0.056449 |
|  | 2.5436 | 2.4453 | 2.5022 | 2.497033 | 0.049353 |
| 23 | 2.5488 | 2.4431 | 2.5128 | 2.501567 | 0.053738 |
|  | 2.5003 | 2.4278 | 2.4761 | 2.468067 | 0.036912 |
| 24 | 2.5193 | 2.4261 | 2.4986 | 2.481333 | 0.04894 |

|  | fliSi 30°C | | | | |
| --- | --- | --- | --- | --- | --- |
| Time (h) | 1 | 2 | 3 | Average | StDev |
| 0 | 0.05 | 0.05 | 0.05 | 0.05 | 0 |
|  | 0.0634 | 0.0646 | 0.0642 | 0.064067 | 0.000611 |
| 1 | 0.0655 | 0.0676 | 0.0688 | 0.0673 | 0.00167 |
|  | 0.0874 | 0.0922 | 0.0957 | 0.091767 | 0.004167 |
| 2 | 0.1386 | 0.146 | 0.1587 | 0.147767 | 0.010166 |
|  | 0.249 | 0.2556 | 0.265 | 0.256533 | 0.008041 |
| 3 | 0.3965 | 0.4074 | 0.4313 | 0.411733 | 0.0178 |
|  | 0.5832 | 0.5947 | 0.6376 | 0.605167 | 0.028671 |
| 4 | 0.794 | 0.8233 | 1.1343 | 0.9172 | 0.188584 |
|  | 1.3711 | 1.3496 | 1.409 | 1.376567 | 0.030075 |
| 5 | 1.5154 | 1.5009 | 1.5784 | 1.531567 | 0.041202 |
|  | 1.6682 | 1.6859 | 1.7509 | 1.701667 | 0.043546 |
| 6 | 1.7863 | 1.8149 | 1.8787 | 1.826633 | 0.047304 |
|  | 1.8983 | 1.9233 | 1.9847 | 1.935433 | 0.04446 |
| 7 | 2.0008 | 2.0199 | 2.0755 | 2.032067 | 0.038808 |
|  | 2.0865 | 2.0971 | 2.1604 | 2.114667 | 0.039959 |
| 8 | 2.1587 | 2.1624 | 2.2191 | 2.180067 | 0.033854 |
|  | 2.2272 | 2.2269 | 2.2805 | 2.244867 | 0.03086 |
| 9 | 2.2831 | 2.2991 | 2.3551 | 2.312433 | 0.037807 |
|  | 2.3578 | 2.3631 | 2.4167 | 2.3792 | 0.032584 |
| 10 | 2.42 | 2.4325 | 2.4559 | 2.436133 | 0.018224 |
|  | 2.4497 | 2.48 | 2.5009 | 2.476867 | 0.025743 |
| 11 | 2.4993 | 2.531 | 2.5742 | 2.534833 | 0.037597 |
|  | 2.5316 | 2.5612 | 2.5682 | 2.553667 | 0.019428 |
| 12 | 2.5412 | 2.5688 | 2.6085 | 2.572833 | 0.033831 |
|  | 2.5261 | 2.5641 | 2.616 | 2.568733 | 0.045129 |
| 13 | 2.5584 | 2.5728 | 2.667 | 2.5994 | 0.058984 |
|  | 2.5811 | 2.6336 | 2.686 | 2.633567 | 0.05245 |
| 14 | 2.5814 | 2.6211 | 2.6811 | 2.627867 | 0.050193 |
|  | 2.5631 | 2.6357 | 2.6805 | 2.626433 | 0.059246 |
| 15 | 2.5906 | 2.6344 | 2.6762 | 2.633733 | 0.042804 |
|  | 2.5862 | 2.6629 | 2.6828 | 2.643967 | 0.051007 |
| 16 | 2.6099 | 2.6557 | 2.7056 | 2.657067 | 0.047865 |
|  | 2.5758 | 2.6156 | 2.6717 | 2.621033 | 0.04818 |
| 17 | 2.5898 | 2.6053 | 2.6546 | 2.616567 | 0.033837 |
|  | 2.594 | 2.6469 | 2.6627 | 2.634533 | 0.035981 |
| 18 | 2.5573 | 2.5954 | 2.6163 | 2.589667 | 0.029915 |
|  | 2.5545 | 2.5838 | 2.6005 | 2.5796 | 0.023286 |
| 19 | 2.5548 | 2.5716 | 2.609 | 2.578467 | 0.027745 |
|  | 2.518 | 2.5544 | 2.5837 | 2.552033 | 0.032914 |
| 20 | 2.5307 | 2.5609 | 2.5867 | 2.559433 | 0.028029 |
|  | 2.5316 | 2.5518 | 2.5661 | 2.549833 | 0.017334 |
| 21 | 2.5155 | 2.5157 | 2.5361 | 2.522433 | 0.011836 |
|  | 2.5152 | 2.511 | 2.5387 | 2.521633 | 0.014929 |
| 22 | 2.4991 | 2.5146 | 2.5375 | 2.517067 | 0.019318 |
|  | 2.4722 | 2.4798 | 2.5069 | 2.4863 | 0.01824 |
| 23 | 2.4652 | 2.4817 | 2.4938 | 2.480233 | 0.014356 |
|  | 2.4489 | 2.4379 | 2.4636 | 2.450133 | 0.012894 |
| 24 | 2.462 | 2.4604 | 2.4644 | 2.462267 | 0.002013 |

|  | fliTi 30°C | | | | |
| --- | --- | --- | --- | --- | --- |
| Time (h) | 1 | 2 | 3 | Average | StDev |
| 0 | 0.05 | 0.05 | 0.05 | 0.05 | 0 |
|  | 0.0767 | 0.0855 | 0.0798 | 0.080667 | 0.004464 |
| 1 | 0.0867 | 0.0915 | 0.0874 | 0.088533 | 0.002593 |
|  | 0.1346 | 0.1426 | 0.1391 | 0.138767 | 0.00401 |
| 2 | 0.2781 | 0.3006 | 0.2952 | 0.2913 | 0.011746 |
|  | 0.4425 | 0.4609 | 0.4509 | 0.451433 | 0.009212 |
| 3 | 0.6441 | 0.6595 | 0.652 | 0.651867 | 0.007701 |
|  | 0.8526 | 0.8626 | 0.8867 | 0.8673 | 0.017529 |
| 4 | 1.1211 | 1.2246 | 1.1898 | 1.1785 | 0.052667 |
|  | 1.4065 | 1.5086 | 1.4846 | 1.466567 | 0.053385 |
| 5 | 1.6374 | 1.6941 | 1.6875 | 1.673 | 0.031007 |
|  | 1.7784 | 1.8136 | 1.8046 | 1.798867 | 0.018287 |
| 6 | 1.88 | 1.9008 | 1.8911 | 1.890633 | 0.010408 |
|  | 1.9354 | 1.9538 | 1.9494 | 1.9462 | 0.009608 |
| 7 | 1.9779 | 1.9923 | 1.9879 | 1.986033 | 0.007379 |
|  | 2.0131 | 2.0147 | 2.0204 | 2.016067 | 0.003837 |
| 8 | 2.0454 | 2.0465 | 2.062 | 2.0513 | 0.009283 |
|  | 2.0972 | 2.0808 | 2.0751 | 2.084367 | 0.011474 |
| 9 | 2.0688 | 2.0795 | 2.0814 | 2.076567 | 0.006793 |
|  | 2.0706 | 2.0902 | 2.0839 | 2.081567 | 0.010006 |
| 10 | 2.0766 | 2.1047 | 2.0717 | 2.084333 | 0.017807 |
|  | 2.0794 | 2.1172 | 2.1151 | 2.1039 | 0.021244 |
| 11 | 2.1167 | 2.1432 | 2.1547 | 2.1382 | 0.019487 |
|  | 2.1695 | 2.1725 | 2.3023 | 2.214767 | 0.075821 |
| 12 | 2.3221 | 2.3182 | 2.4608 | 2.367033 | 0.081228 |
|  | 2.4375 | 2.415 | 2.5096 | 2.454033 | 0.04942 |
| 13 | 2.5159 | 2.4698 | 2.5574 | 2.514367 | 0.04382 |
|  | 2.5758 | 2.5035 | 2.6032 | 2.560833 | 0.051508 |
| 14 | 2.5554 | 2.5195 | 2.6005 | 2.558467 | 0.040587 |
|  | 2.5806 | 2.523 | 2.6045 | 2.569367 | 0.041895 |
| 15 | 2.5785 | 2.5302 | 2.6252 | 2.577967 | 0.047502 |
|  | 2.5851 | 2.5345 | 2.6287 | 2.582767 | 0.047143 |
| 16 | 2.5766 | 2.5581 | 2.6232 | 2.585967 | 0.033546 |
|  | 2.5494 | 2.5128 | 2.5669 | 2.543033 | 0.027606 |
| 17 | 2.5567 | 2.5025 | 2.5544 | 2.537867 | 0.03065 |
|  | 2.5538 | 2.5049 | 2.5707 | 2.543133 | 0.034172 |
| 18 | 2.5615 | 2.4831 | 2.5406 | 2.5284 | 0.040599 |
|  | 2.5398 | 2.464 | 2.5141 | 2.505967 | 0.038549 |
| 19 | 2.5289 | 2.4497 | 2.5188 | 2.499133 | 0.043107 |
|  | 2.5137 | 2.4224 | 2.4836 | 2.473233 | 0.046524 |
| 20 | 2.521 | 2.4211 | 2.4927 | 2.478267 | 0.05149 |
|  | 2.4949 | 2.4081 | 2.4889 | 2.463967 | 0.048475 |
| 21 | 2.4637 | 2.371 | 2.4573 | 2.430667 | 0.051772 |
|  | 2.474 | 2.3792 | 2.4619 | 2.438367 | 0.051596 |
| 22 | 2.449 | 2.3654 | 2.4797 | 2.431367 | 0.059155 |
|  | 2.4309 | 2.3417 | 2.4838 | 2.4188 | 0.071819 |
| 23 | 2.3992 | 2.3175 | 2.4902 | 2.4023 | 0.086392 |
|  | 2.3831 | 2.3099 | 2.4615 | 2.384833 | 0.075815 |
| 24 | 2.3784 | 2.3125 | 2.5001 | 2.397 | 0.095173 |

|  | fliYi 30°C | | | | |
| --- | --- | --- | --- | --- | --- |
| Time (h) | 1 | 2 | 3 | Average | StDev |
| 0 | 0.05 | 0.05 | 0.05 | 0.05 | 0 |
|  | 0.0647 | 0.0652 | 0.0648 | 0.0649 | 0.000265 |
| 1 | 0.0641 | 0.0642 | 0.0649 | 0.0644 | 0.000436 |
|  | 0.0825 | 0.085 | 0.0867 | 0.084733 | 0.002113 |
| 2 | 0.1479 | 0.1527 | 0.1544 | 0.151667 | 0.003371 |
|  | 0.2564 | 0.2617 | 0.2651 | 0.261067 | 0.004384 |
| 3 | 0.4556 | 0.4614 | 0.4683 | 0.461767 | 0.006358 |
|  | 0.6624 | 0.6701 | 0.6859 | 0.6728 | 0.01198 |
| 4 | 0.8356 | 0.9282 | 0.9529 | 0.905567 | 0.061839 |
|  | 1.3346 | 1.3106 | 1.3031 | 1.3161 | 0.016454 |
| 5 | 1.6199 | 1.5781 | 1.5693 | 1.5891 | 0.027034 |
|  | 1.7783 | 1.7424 | 1.7562 | 1.758967 | 0.018109 |
| 6 | 1.8631 | 1.8543 | 1.8855 | 1.867633 | 0.016086 |
|  | 1.9259 | 1.9123 | 1.9607 | 1.932967 | 0.024962 |
| 7 | 1.9745 | 1.9717 | 2.0133 | 1.9865 | 0.023252 |
|  | 2.0049 | 2.021 | 2.0591 | 2.028333 | 0.027834 |
| 8 | 2.041 | 2.055 | 2.1133 | 2.069767 | 0.038345 |
|  | 2.0748 | 2.086 | 2.14 | 2.100267 | 0.034863 |
| 9 | 2.1035 | 2.1074 | 2.2122 | 2.141033 | 0.061663 |
|  | 2.1046 | 2.1464 | 2.311 | 2.187333 | 0.109119 |
| 10 | 2.0782 | 2.2431 | 2.3208 | 2.214033 | 0.123884 |
|  | 2.1024 | 2.2836 | 2.3222 | 2.236067 | 0.117357 |
| 11 | 2.1378 | 2.3151 | 2.3837 | 2.278867 | 0.126891 |
|  | 2.263 | 2.3262 | 2.3519 | 2.3137 | 0.045749 |
| 12 | 2.4435 | 2.4056 | 2.4087 | 2.419267 | 0.021044 |
|  | 2.4928 | 2.4502 | 2.4526 | 2.4652 | 0.023932 |
| 13 | 2.5667 | 2.4993 | 2.491 | 2.519 | 0.041517 |
|  | 2.6365 | 2.5741 | 2.5383 | 2.582967 | 0.049697 |
| 14 | 2.6185 | 2.5875 | 2.579 | 2.595 | 0.020791 |
|  | 2.6591 | 2.6491 | 2.6118 | 2.64 | 0.024928 |
| 15 | 2.6556 | 2.6636 | 2.6537 | 2.657633 | 0.005254 |
|  | 2.6601 | 2.6796 | 2.6731 | 2.670933 | 0.009929 |
| 16 | 2.6842 | 2.6806 | 2.6849 | 2.683233 | 0.002307 |
|  | 2.6256 | 2.6832 | 2.6623 | 2.657033 | 0.029159 |
| 17 | 2.6142 | 2.6415 | 2.6495 | 2.635067 | 0.018508 |
|  | 2.6356 | 2.6351 | 2.6469 | 2.6392 | 0.006673 |
| 18 | 2.623 | 2.6255 | 2.6214 | 2.6233 | 0.002066 |
|  | 2.5898 | 2.6022 | 2.5786 | 2.5902 | 0.011805 |
| 19 | 2.5928 | 2.5886 | 2.5898 | 2.5904 | 0.002163 |
|  | 2.5676 | 2.562 | 2.5833 | 2.570967 | 0.011042 |
| 20 | 2.5774 | 2.5766 | 2.5802 | 2.578067 | 0.00189 |
|  | 2.5699 | 2.5651 | 2.5686 | 2.567867 | 0.002483 |
| 21 | 2.5383 | 2.5282 | 2.5307 | 2.5324 | 0.00526 |
|  | 2.5376 | 2.5411 | 2.5331 | 2.537267 | 0.00401 |
| 22 | 2.5324 | 2.5313 | 2.4938 | 2.519167 | 0.021975 |
|  | 2.5286 | 2.5379 | 2.4804 | 2.515633 | 0.030865 |
| 23 | 2.5226 | 2.5304 | 2.4618 | 2.504933 | 0.037558 |
|  | 2.5005 | 2.5057 | 2.4374 | 2.4812 | 0.038021 |
| 24 | 2.5063 | 2.5028 | 2.4446 | 2.484567 | 0.034656 |
